# Xtrapol8 enables automatic elucidation of low-occupancy intermediate-states in crystallographic studies

**DOI:** 10.1038/s42003-022-03575-7

**Published:** 2022-06-29

**Authors:** Elke De Zitter, Nicolas Coquelle, Paula Oeser, Thomas R. M. Barends, Jacques-Philippe Colletier

**Affiliations:** 1 Univ. Grenoble Alpes, CEA, CNRS, Institut de Biologie Structurale, 38000 Grenoble, France; 2 European Synchrotron Radiation Facility (ESRF), BP 220, 38043 Grenoble, France; 3 Max-Planck-Institut für medizinische Forschung, Jahnstrasse 29, 69120 Heidelberg, Germany

**Keywords:** X-ray crystallography, Data processing, Software

## Abstract

Unstable states studied in kinetic, time-resolved and ligand-based crystallography are often characterized by a low occupancy, which hinders structure determination by conventional methods. To automatically extract structural information pertaining to these states, we developed Xtrapol8, a program which (i) applies various flavors of Bayesian-statistics weighting to generate the most informative Fourier difference maps; (ii) determines the occupancy of the intermediate states by use of methods *hitherto* not available; (iii) calculates extrapolated structure factors using the various proposed formalisms while handling the issue of negative structure factor amplitudes, and (iv) refines the corresponding structures in real and reciprocal-space. The use of Xtrapol8 could accelerate data processing in kinetic and time-resolved crystallographic studies, and as well foster the identification of drug-targetable states in ligand-based crystallography.

## Abstract (web summary)

The software Xtrapol8 extracts low occupancy states and can thereby accelerate and facilitate data processing in kinetic, timeresolved and ligand-based crystallographic studies.

## Introduction

Once reserved to a handful of proteins and specialized laboratories, time-resolved and kinetic crystallography (TRX, KX) are on the verge of widespread adoption. This momentum is owed mostly to the advent of room-temperature serial crystallography, pioneered at X-ray free electron lasers (XFEL)^1^ yet swiftly implemented at synchrotrons where ease of access and a larger user-base hold promise for groundbreaking studies^2^. A main limitation of TRX and KX remains that full occupancy of the triggered state is hardly ever attained in the crystalline macromolecule, resulting in co-existence with the reference state. Low occupancy may also poison data collected from crystalline ligand-protein complexes, obscuring ligand identification and conformational changes undergone by the protein upon binding.

Based on the assumption that structure factor phases hardly vary upon reaction initiation and progression, and provided that the two datasets are isomorphous, the calculation of Fourier difference electron density maps is a convenient means to highlight the largest structural differences between a reference and a triggered state dataset^3,4^:

1 $$\left|{F}_{{{{{{\rm{obs}}}}}}}^{{{{{{\rm{triggered}}}}}}}-{F}_{{{{{{\rm{obs}}}}}}}^{{{{{{\rm{reference}}}}}}}\right|,{\varphi }_{{{{{{\rm{calc}}}}}}}^{{{{{{\rm{reference}}}}}}}$$

This assumption holds true for the vast majority of TRX and KX experiments, where only limited conformational changes are observed, and as well for binding of a small molecule to a crystalline protein. It may further remain an acceptable approximation for larger structural changes in which a substantial number of atoms in the unit cell are involved, provided that the isomorphism between the two dataset remains high^5^ (e.g., ref. ^6^). The information content of Fourier difference maps can be improved by Bayesian-statistics weighting of structure factor amplitude (SFA) differences^7,8^, yet these maps can be featureless when structural changes or the triggered-state occupancy are small, or if a mixture of triggered states accumulates^9^, whose overlaying positive and negative difference peaks cancel each other. Furthermore, Fourier difference maps may allow modeling of the triggered state structure, but not its refinement, limiting both insights and reproducibility. By use of extrapolation methods, whereby SFA differences are inversely scaled to the occupancy of the triggered state and summed with reference SFAs, the hypothetical SFAs for the triggered state present at full occupancy can be estimated, enabling both refinement of the triggered state structure, and to identify potential co-existing intermediate states:

2 $${F}_{{{\mbox{extrapolated}}}}=w\times \alpha \times \left({F}_{{{{{{\rm{obs}}}}}}}^{{{{{{\rm{triggered}}}}}}}-{F}_{{{{{{\rm{obs}}}}}}}^{{{{{{\rm{reference}}}}}}}\right)+{F}_{{{\mbox{additional}}}}$$

where α is the reciprocal occupancy of the triggered state (α = 1/occupancy) and *w* a potential weighting factor. With the advent of serial crystallography, and the consequential blooming of TRX studies, the demand for extrapolation methods has risen, but these remain obscure to the vast majority of crystallographers for three main reasons. Firstly, the chosen occupancy is determined but hardly ever justified in absence of methods to estimate the triggered state occupancy based on crystallographic data only. Secondly, each lab resorting to structure factor extrapolation makes use of its own library of custom-written scripts, generally assembled over years of practice in TRX and KX, making results not easily reproducible. Thirdly, various flavors of extrapolation exist whereby users have the possibility to exploit Bayesian-statistics to downweigh SFA difference outliers, refine phases from the reference state structure against extrapolated SFAs (ESFAs), and recalculate the figure of merit of these phases prior to generation of extrapolated maps. Here, we introduce Xtrapol8, a new software aimed at resolving these issues, and discuss its design and usage. Briefly, provided a triggered state dataset, and a reference dataset and model, Xtrapol8 firstly calculates a weighted Fourier difference electron density map to unbiasedly visualize differences between the reference and triggered state. Secondly, Xtrapol8 computes optionally-weighted ESFAs, and carries out real-space and reciprocal-space refinements in an automated fashion. Finally, Xtrapol8 provides estimates of the triggered state occupancy via two independent methods *hitherto* not available. The *difference-map* method estimates the occupancy based on difference electron density maps, whereas the *distance-analysis* method relies on the automatically refined (reciprocal-space and realspace) structures. We showcase Xtrapol8’s utility by application to recently published KX data collected on the photoconvertible fluorescent protein mEos4b^10^. In the Supplementary Information we further evaluate the versatility of Xtrapol8 by revisiting a variety of previously-published challenging TRX or KX studies.

## Results and discussion

**Fig. 1:**
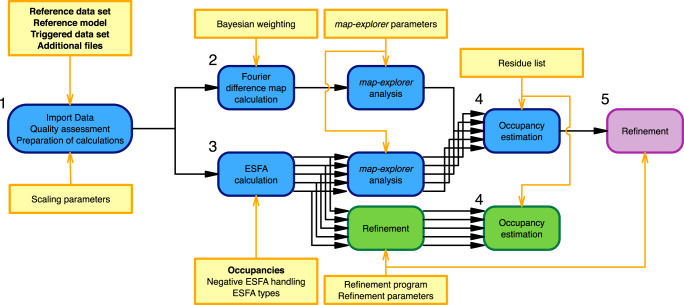
Design of Xtrapol8. Xtrapol8 roadmap. The four main steps followed by Xtrapol8 are depicted in blue. User input are highlighted by yellow boxes, with obligatory input further highlighted in bold. Steps specific to the ‘*fast-and-furious*’ (default options: *q*-weighting of difference map and ESFAs, rescue of negative ESFAs using the *truncate* method, occupancy determination based on the *difference-map* method; additional step 5: refinement in both reciprocal-space and real-space at the automatically determined occupancy) and ‘*calm-and-curious*’ modes are boxed in purple and green, respectively.

Written in python, Xtrapol8 requires only the CCP4^11^ and Phenix^12^ suites to run. Only standard packages and the cctbx toolbox^13^ are used, which are automatically loaded from the phenix.python environment. This facilitates usage as well as transfer between laboratory computers and data collection centers (XFELs, synchrotrons). Fig. 1 shows Xtrapol8’s overall outline and recommended usage. The minimal required input are two data files in mtz or mmcif format, associated to the reference (ground, native, apoprotein) and the triggered (derivative, ligand-bound, intermediate, excited) state, respectively; a model associated to the reference state in pdb or mmcif format; additional files required for proper handling of ligands (such as crystallographic information files, or custom modification files), and a list of possible occupancies for the triggered state (Fig. 1). Depending on the user’s experience, many additional parameters can be adjusted including resolution cutoffs, scaling parameters, weighting schemes, and parameters for difference map exploration or structure refinement.

Xtrapol8 takes four main steps when run in the ‘*calm-and-curious’* mode (Fig. 1): (1) Reading of input files and quality assessment, and preparation of the files needed for the next steps; (2) Calculation of the optionally-weighted Fourier difference map, integration of peaks and assignment to reference model atoms; (3) Calculation of ESFAs using a variety of formalisms for a user-defined range of possible occupancies, integration of peaks in the extrapolated difference maps and assignment to reference model atoms, and optional automatic structure refinements; (4) Estimation of the triggered state occupancy and assignment of the most likely ESFAs and electron density maps for the triggered state structure. The program can also be run in a ‘*fast-and-furious’* mode, in which the refinements in steps 3 are omitted and only performed in a fifth additional step at the automatically determined occupancy of the triggered state. In the ‘*Fo-Fo map only*’ mode, only the first and second step are carried out.

### Graphical user interface

**Fig. 2:**
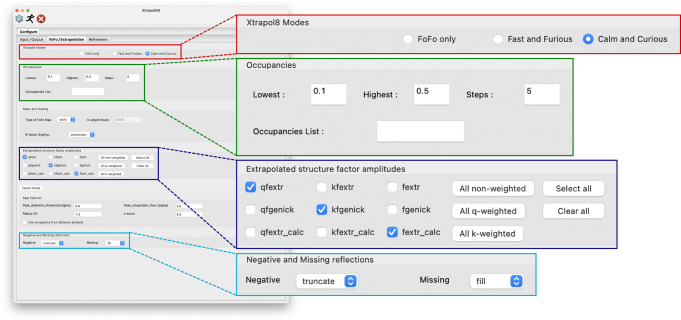
The graphical user interface allows facilitated parameter control. Panel of the GUI in which the user can alter the options concerning the Fourier difference maps and ESFAs, with zoom-in on some input fields. Details on all input and output tabs and panels is provided in Supplementary Figs. 1–5.

Xtrapol8 can be controlled through the command line or via a graphical user interface (XtrapolG8) written in wxPython as a frontend to the command-line version. The ‘*Configure’* tab features three input panels arranged according to the different steps (*Input/Output*, *FoFo/Extrapolation*, *Refinement*) with a selection of options and parameters that are hidden/shown depending on the user-selected expertise level and Xtrapol8 mode (*Fo-Fo map only*, *fast-and-furious*, *calm-and-curious*; Fig. 2, Supplementary Figs. 1–4). The ‘*Results’* tab also consists of three panels. The first panel shows the progression of the Xtrapol8 run by tailing the log file. The second panel displays the general output figures, e.g., the plots of *R*_iso_ and CC_iso_ (see below) as a function of resolution, of the fraction of negative ESFAs as a function of intermediate state occupancy, or the results of the occupancy determinations and refinements (Supplementary Fig. 5a). The last panel shows plots specific to a given type of ESFAs and occupancy, accessible through two dropdown menus, e.g., the plots of the number of negative ESFAs or of ESFA signal-to-noise ratio as a function of resolution. Coot can be directly opened from XtrapolG8 and will load the Fourier difference and extrapolated maps, and the refinements’ output (Supplementary Fig. 5b, c). For the automatically determined occupancy, an all-atom distance difference matrix is also computed between the refined extrapolated-structure and the reference model, whereby the plotted value for each residue corresponds to the sum of the changes in distance for all constitutive atoms.

### Data handling and quality assessment

The input data files should contain merged data in the form of SFAs or intensities (Supplementary Methods). In the case where only the latter are available, they will be converted into SFAs using the French-Wilson algorithm^14^ in truncate (CCP4)^15^. Afterwards, pointless^15^ will be run to verify consistent indexing between the triggered and reference datasets, and if needed, will be used to reindex the triggered dataset in respect to the reference dataset. The two datasets are then trimmed so as to retain only reflections present in both sets of observed data and lying within the optionally user-defined resolution limits. In the case where no resolution limit is defined, the program will use all data present in the input data files.

Prior to difference map and ESFA calculations, the model-derived calculated SFAs are scaled anisotropically to the reference data using mmtbx.fmodel^16,17^ while scaling of the triggered to the reference data is performed anisotropically or isotropically by scaleit^11^. At this step, statistical indicators informing on the isomorphism between the input data sets are calculated, which inform users on the probability that both sensible Fourier difference maps and ESFAs can be calculated. Specifically, the *R*-factor (*R*_iso_) between the two datasets is calculated using:

3 $${R}_{{{\mbox{iso}}}}=\frac{\sum \left|{F}_{{{{{{\rm{obs}}}}}}}^{{{{{{\rm{reference}}}}}}}-{F}_{{{{{{\rm{obs}}}}}}}^{{{{{{\rm{triggered}}}}}}}\right|}{\sum \left|{F}_{{{{{{\rm{obs}}}}}}}^{{{{{{\rm{reference}}}}}}}+{F}_{{{{{{\rm{obs}}}}}}}^{{{{{{\rm{triggered}}}}}}}\right|/2}$$

where ${F}_{{{{{{\rm{obs}}}}}}}^{{{{{{\rm{reference}}}}}}}$ and ${F}_{{{{{{\rm{obs}}}}}}}^{{{{{{\rm{triggered}}}}}}}$ are the SFAs of the reference and triggered states, respectively. An overall R_iso_ value lower than 0.10 indicates strong isomorphism between the two data sets, but values up to 0.25 can still lead to useful difference and extrapolated electron density maps^4^. The isomorphism-indicating correlation coefficient (cc_iso_) is calculated using:

4 $${{{\mbox{cc}}}}_{{{\mbox{iso}}}}=\frac{\sum \left[\left({F}_{{{{{{\rm{obs}}}}}}}^{{{{{{\rm{reference}}}}}}}-\overline{{F}_{{{{{{\rm{obs}}}}}}}^{{{{{{\rm{reference}}}}}}}}\right)\times \left({F}_{{{{{{\rm{obs}}}}}}}^{{{{{{\rm{triggered}}}}}}}-\overline{{F}_{{{{{{\rm{obs}}}}}}}^{{{{{{\rm{triggered}}}}}}}}\right)\right]}{\sqrt{\sum {\left({F}_{{{{{{\rm{obs}}}}}}}^{{{{{{\rm{reference}}}}}}}-\overline{{F}_{{{{{{\rm{obs}}}}}}}^{{{{{{\rm{reference}}}}}}}}\right)}^{2}\times \sum {\left({F}_{{{{{{\rm{obs}}}}}}}^{{{{{{\rm{triggered}}}}}}}-\overline{{F}_{{{{{{\rm{obs}}}}}}}^{{{{{{\rm{triggered}}}}}}}}\right)}^{2}}}$$

### Experimental Fourier difference maps calculation

On the assumption that the structural differences between the reference and triggered state(s) are small—i.e., only a few specific atoms change position, with no rotation or translation of the molecule in the unit cell—the phases of the triggered and the reference states hardly differ, which enables investigators to extract information on the triggered state by calculation of a Fourier difference map (Eq. 1). A simple subtraction between the two sets of SFAs gives a similar weight to all of them, regardless of their measurement precision. Ursby and Bourgeois^7^ suggested the use of *q*-weighting, whereby the magnitude of the difference between SFAs and the relative errors on their measurements are used to downweigh unlikely SFA differences. The use of Bayesian statistics to improve Fourier difference maps was also proposed by Terwilliger and Berendzen^18^. Later, Ren et al. introduced a simpler weighting scheme^8^, to which we refer to as *k*-weighting in analogy to *q*-weighting. In Xtrapol8, the user has the choice to calculate *q*-weighted, *k*-weighted or non-weighted Fourier difference maps, but the default is set to *q*-weighting. In the *k*-weighting scheme, the extent to which outliers are downweighed can be adjusted by an additional parameter, that has been set to 0.05^19^ or 1.0^8,20^ depending on reports, with the first one being the default in Xtrapol8.

### Difference map analysis

An important feature of Xtrapol8 is the *map-explorer* module, which will search for peaks in difference maps, integrate and assign them to the closest reference model atom within a given radius, and generate an unbiased list of residues whose positions change relative to those in the reference model. Based on this list, the residues with the highest integrated peak volumes can be used to evaluate the occupancy of the triggered state in a later stage of the program. The stringency in the selection of difference peaks can be adjusted—not only in terms of the maximal and minimal heights for peak selection and integration, respectively, but as well in terms of Z-score filtering and search radius. Default values for first three parameters are ± 4 r.m.s.d., ± 3 r.m.s.d. and 2, while the search radius is defaulted to the highest resolution.

### Extrapolated structure factor amplitudes (ESFAs)

Extrapolated structure factor amplitudes are estimates of the SFAs that the triggered state would have given rise to if it had been present at full occupancy in the crystal. The general formula for calculating ESFAs is presented in Eq. 2. The weighting factor *w* and the F_additional_ term have found different interpretations in the literature, explaining that various extrapolation approaches exist. Firstly, *F*_additional_ was initially derived from the reference model (i.e., ${F}_{{{{{{\rm{model}}}}}}}^{{{{{{\rm{reference}}}}}}}$ or ${F}_{{{{{{\rm{calc}}}}}}}^{{{{{{\rm{reference}}}}}}}$ depending on whether or not bulk-solvent modeling is included)^18^, but use of measured reference state SFAs (${F}_{{{{{{\rm{obs}}}}}}}^{{{{{{\rm{reference}}}}}}}$) was later put forward by Genick and co-workers^5,21^. Whether the ${F}_{{{{{{\rm{obs}}}}}}}^{{{{{{\rm{reference}}}}}}}$ should be preferred to ${F}_{{{{{{\rm{model}}}}}}}^{{{{{{\rm{reference}}}}}}}$ is still under debate^22^, illustrating that it likely depends on the experimental case. Secondly, one can question whether or not the *q/k*-weighting scheme used in Fourier difference map calculations to reduce the weight of uncertain SFA differences should also be applied in the calculation of ESFAs (weighting factor *w*)^23–25^, leading to yet another nuance. Finally and perhaps more subtly, initial extrapolated electron density maps are being calculated using the phases of the reference model, but the associated figure of merit (*m*) can be chosen to represent the phase agreement between the ESFAs and reference state model, or originate from the reference state^5,21^. As the latter has been proposed by Genick et al.^5^, we refer to these as the (*q/k*)Fgenick ESFA type. Overall, this leads to nine different types of ESFAs and corresponding extrapolated maps (Table 1), reflecting the variety of approaches that have been followed depending on the experimental case and laboratory since introduction of extrapolation methods.

**Table 1:** Types of ESFAs calculated by Xtrapol8 and corresponding map coefficients.

Type	Extrapolated structure factors	Map coefficients	
		2*F*_extrapolated_ − *F*_calc_ type	*F*_extrapolated_ − *F*_calc_ type
*F*_extr_	$\,{F}_{{{{{{\rm{extr}}}}}}}=\,{{{{{\rm{\alpha }}}}}}\,\times \left({F}_{{{{{{\rm{obs}}}}}}}^{{{{{{\rm{trig}}}}}}}-{F}_{{{{{{\rm{obs}}}}}}}^{{{{{{\rm{ref}}}}}}}\right)+{F}_{{{{{{\rm{obs}}}}}}}^{{{{{{\rm{ref}}}}}}}$	$2{m}\left|{F}_{{{{{{\rm{extr}}}}}}}\right|-{D}\left|{F}_{{{{{{\rm{calc}}}}}}}^{{{{{{\rm{ref}}}}}}}\right|,\,{{{{{{\rm{\varphi }}}}}}}_{{{{{{\rm{calc}}}}}}}^{{{{{{\rm{ref}}}}}}}$	${m}\left|{F}_{{{{{{\rm{extr}}}}}}}\right|-{D}\left|{F}_{{{{{{\rm{calc}}}}}}}^{{{{{{\rm{ref}}}}}}}\right|,\,{{{{{{\rm{\varphi }}}}}}}_{{{{{{\rm{calc}}}}}}}^{{{{{{\rm{ref}}}}}}}$
*qF*_extr_	${q}{F}_{{{{{{\rm{extr}}}}}}}=\,{{{{{\rm{\alpha }}}}}}\,\times \frac{{q}}{{{\langle }}{q}{{\rangle }}}\,\times \left({F}_{{{{{{\rm{obs}}}}}}}^{{{{{{\rm{trig}}}}}}}-{F}_{{{{{{\rm{obs}}}}}}}^{{{{{{\rm{ref}}}}}}}\right)+{F}_{{{{{{\rm{obs}}}}}}}^{{{{{{\rm{ref}}}}}}}$	$2{m}\left|{q}{F}_{{{{{{\rm{extr}}}}}}}\right|-{D}\left|{F}_{{{{{{\rm{calc}}}}}}}^{{{{{{\rm{ref}}}}}}}\right|,\,{{{{{{\rm{\varphi }}}}}}}_{{{{{{\rm{calc}}}}}}}^{{{{{{\rm{ref}}}}}}}$	${m}\left|{{{{{{\rm{qF}}}}}}}_{{{{{{\rm{extr}}}}}}}\right|-{D}\left|{F}_{{{{{{\rm{calc}}}}}}}^{{{{{{\rm{ref}}}}}}}\right|,\,{{{{{{\rm{\varphi }}}}}}}_{{{{{{\rm{calc}}}}}}}^{{{{{{\rm{ref}}}}}}}$
*kF*_extr_	${q}{F}_{{{{{{\rm{extr}}}}}}}=\,{{{{{\rm{\alpha }}}}}}\,\times \frac{{{{{{\rm{k}}}}}}}{{{\langle }}{{{{{\rm{k}}}}}}{{\rangle }}}\,\times \left({F}_{{{{{{\rm{obs}}}}}}}^{{{{{{\rm{trig}}}}}}}-{F}_{{{{{{\rm{obs}}}}}}}^{{{{{{\rm{ref}}}}}}}\right)+{F}_{{{{{{\rm{obs}}}}}}}^{{{{{{\rm{ref}}}}}}}$	$2{m}\left|{{{{{{\rm{kF}}}}}}}_{{{{{{\rm{extr}}}}}}}\right|-{D}\left|{F}_{{{{{{\rm{calc}}}}}}}^{{{{{{\rm{ref}}}}}}}\right|,\,{{{{{{\rm{\varphi }}}}}}}_{{{{{{\rm{calc}}}}}}}^{{{{{{\rm{ref}}}}}}}$	${m}\left|{{{{{\rm{k}}}}}}{F}_{{{{{{\rm{extr}}}}}}}\right|-{D}\left|{F}_{{{{{{\rm{calc}}}}}}}^{{{{{{\rm{ref}}}}}}}\right|,\,{{{{{{\rm{\varphi }}}}}}}_{{{{{{\rm{calc}}}}}}}^{{{{{{\rm{ref}}}}}}}$
*F*_genick_	$\,{F}_{{{{{{\rm{genick}}}}}}}=\,{{{{{\rm{\alpha }}}}}}\,\times \left({F}_{{{{{{\rm{obs}}}}}}}^{{{{{{\rm{trig}}}}}}}-\,{F}_{{{{{{\rm{obs}}}}}}}^{{{{{{\rm{ref}}}}}}}\right)+{F}_{{{{{{\rm{obs}}}}}}}^{{{{{{\rm{ref}}}}}}}$	${{m}}^{{{{{{\rm{ref}}}}}}}\left|{F}_{{{{{{\rm{genick}}}}}}}\right|,\,{{{{{{\rm{\varphi }}}}}}}_{{{{{{\rm{calc}}}}}}}^{{{{{{\rm{ref}}}}}}}$	${{m}}^{{{{{{\rm{ref}}}}}}}\left|{F}_{{{{{{\rm{genick}}}}}}}\right|-{D}\left|{F}_{{{{{{\rm{calc}}}}}}}^{{{{{{\rm{ref}}}}}}}\right|,\,{{{{{{\rm{\varphi }}}}}}}_{{{{{{\rm{calc}}}}}}}^{{{{{{\rm{ref}}}}}}}$
*qF*_genick_	${q}{F}_{{{{{{\rm{genick}}}}}}}=\,{{{{{\rm{\alpha }}}}}}\,\times \frac{{q}}{{{\langle }}{q}{{\rangle }}}\,\times \left({F}_{{{{{{\rm{obs}}}}}}}^{{{{{{\rm{trig}}}}}}}-\,{F}_{{{{{{\rm{obs}}}}}}}^{{{{{{\rm{ref}}}}}}}\right)+{F}_{{{{{{\rm{obs}}}}}}}^{{{{{{\rm{ref}}}}}}}$	${{m}}^{{{{{{\rm{ref}}}}}}}\left|{q}{F}_{{{{{{\rm{genick}}}}}}}\right|,\,{{{{{{\rm{\varphi }}}}}}}_{{{{{{\rm{calc}}}}}}}^{{{{{{\rm{ref}}}}}}}$	${{m}}^{{{{{{\rm{ref}}}}}}}\left|{{{{{{\rm{qF}}}}}}}_{{{{{{\rm{genick}}}}}}}\right|-{D}\left|{F}_{{{{{{\rm{calc}}}}}}}^{{{{{{\rm{ref}}}}}}}\right|,\,{{{{{{\rm{\varphi }}}}}}}_{{{{{{\rm{calc}}}}}}}^{{{{{{\rm{ref}}}}}}}$
*kF*_genick_	${q}{F}_{{{{{{\rm{genick}}}}}}}=\,{{{{{\rm{\alpha }}}}}}\,\times \frac{{{{{{\rm{k}}}}}}}{{{\langle }}{{{{{\rm{k}}}}}}{{\rangle }}}\,\times \left({F}_{{{{{{\rm{obs}}}}}}}^{{{{{{\rm{trig}}}}}}}-\,{F}_{{{{{{\rm{obs}}}}}}}^{{{{{{\rm{ref}}}}}}}\right)+{F}_{{{{{{\rm{obs}}}}}}}^{{{{{{\rm{ref}}}}}}}$	${{m}}^{{{{{{\rm{ref}}}}}}}\left|{{{{{{\rm{kF}}}}}}}_{{{{{{\rm{genick}}}}}}}\right|,\,{{{{{{\rm{\varphi }}}}}}}_{{{{{{\rm{calc}}}}}}}^{{{{{{\rm{ref}}}}}}}$	${{m}}^{{{{{{\rm{ref}}}}}}}\left|{{{{{\rm{k}}}}}}{F}_{{{{{{\rm{genick}}}}}}}\right|-{D}\left|{F}_{{{{{{\rm{calc}}}}}}}^{{{{{{\rm{ref}}}}}}}\right|,\,{{{{{{\rm{\varphi }}}}}}}_{{{{{{\rm{calc}}}}}}}^{{{{{{\rm{ref}}}}}}}$
*F*_extr_calc_	${F}_{{{{{{\rm{extr}}}}}}\_{{{{{\rm{calc}}}}}}}=\,{{{{{\rm{\alpha }}}}}}\,\times \left({F}_{{{{{{\rm{obs}}}}}}}^{{{{{{\rm{trig}}}}}}}-\,{F}_{{{{{{\rm{obs}}}}}}}^{{{{{{\rm{ref}}}}}}}\right)+{F}_{{{{{{\rm{model}}}}}}}^{{{{{{\rm{ref}}}}}}}$	$2{m}\left|{F}_{{{{{{\rm{extr}}}}}}\_{{{{{\rm{calc}}}}}}}\right|-{D}\left|{F}_{{{{{{\rm{calc}}}}}}}^{{{{{{\rm{ref}}}}}}}\right|,\,{{{{{{\rm{\varphi }}}}}}}_{{{{{{\rm{calc}}}}}}}^{{{{{{\rm{ref}}}}}}}$	${m}\left|{F}_{{{{{{\rm{extr}}}}}}\_{{{{{\rm{calc}}}}}}}\right|-{D}\left|{F}_{{{{{{\rm{calc}}}}}}}^{{{{{{\rm{ref}}}}}}}\right|,\,{{{{{{\rm{\varphi }}}}}}}_{{{{{{\rm{calc}}}}}}}^{{{{{{\rm{ref}}}}}}}$
*qF*_extr_calc_	${q}{F}_{{{{{{\rm{extr}}}}}}\_{{{{{\rm{calc}}}}}}}=\,{{{{{\rm{\alpha }}}}}}\,\times \frac{{q}}{{{\langle }}{q}{{\rangle }}}\,\times \left({F}_{{{{{{\rm{obs}}}}}}}^{{{{{{\rm{trig}}}}}}}\,{-{{{{{\rm{F}}}}}}}_{{{{{{\rm{obs}}}}}}}^{{{{{{\rm{ref}}}}}}}\right)+{F}_{{{{{{\rm{model}}}}}}}^{{{{{{\rm{ref}}}}}}}$	$2{m}\left|{{{{{{\rm{qF}}}}}}}_{{{{{{\rm{extr}}}}}}\_{{{{{\rm{calc}}}}}}}\right|-{D}\left|{F}_{{{{{{\rm{calc}}}}}}}^{{{{{{\rm{ref}}}}}}}\right|,\,{{{{{{\rm{\varphi }}}}}}}_{{{{{{\rm{calc}}}}}}}^{{{{{{\rm{ref}}}}}}}$	${m}\left|{{{{{{\rm{qF}}}}}}}_{{{{{{\rm{extr}}}}}}\_{{{{{\rm{calc}}}}}}}\right|-{D}\left|{F}_{{{{{{\rm{calc}}}}}}}^{{{{{{\rm{ref}}}}}}}\right|,\,{{{{{{\rm{\varphi }}}}}}}_{{{{{{\rm{calc}}}}}}}^{{{{{{\rm{ref}}}}}}}$
*kF*_extr_calc_	${{{{{\rm{kF}}}}}}_{{{{{{\rm{extr}}}}}}\_{{{{{\rm{calc}}}}}}}=\,{{{{{\rm{\alpha }}}}}}\,\times \frac{{{{{{\rm{k}}}}}}}{{{\langle }}{{{{{\rm{k}}}}}}{{\rangle }}}\,\times \left({F}_{{{{{{\rm{obs}}}}}}}^{{{{{{\rm{trig}}}}}}}-\,{F}_{{{{{{\rm{obs}}}}}}}^{{{{{{\rm{ref}}}}}}}\right)+{F}_{{{{{{\rm{model}}}}}}}^{{{{{{\rm{ref}}}}}}}$	$2{m}\left|{{{{{{\rm{kF}}}}}}}_{{{{{{\rm{extr}}}}}}\_{{{{{\rm{calc}}}}}}}\right|-{D}\left|{F}_{{{{{{\rm{calc}}}}}}}^{{{{{{\rm{ref}}}}}}}\right|,\,{{{{{{\rm{\varphi }}}}}}}_{{{{{{\rm{calc}}}}}}}^{{{{{{\rm{ref}}}}}}}$	${m}\left|{{{{{{\rm{kF}}}}}}}_{{{{{{\rm{extr}}}}}}\_{{{{{\rm{calc}}}}}}}\right|-{D}\left|{F}_{{{{{{\rm{calc}}}}}}}^{{{{{{\rm{ref}}}}}}}\right|,\,{{{{{{\rm{\varphi }}}}}}}_{{{{{{\rm{calc}}}}}}}^{{{{{{\rm{ref}}}}}}}$

*α* = 1/occupancy; ${F}_{{{{{{\rm{obs}}}}}}}^{{{{{{\rm{trig}}}}}}}$, ${F}_{{{{{{\rm{obs}}}}}}}^{{{{{{\rm{ref}}}}}}}$: observed structure factors amplitudes associated to triggered and reference state, respectively; ${F}_{{{{{{\rm{model}}}}}}}^{{{{{{\rm{ref}}}}}}}$ and ${F}_{{{{{{\rm{calc}}}}}}}^{{{{{{\rm{ref}}}}}}}$: calculated structure factors associated to reference state, including bulk-solvent modeling;${\varphi }_{{{{{{\rm{calc}}}}}}}^{{{{{{\rm{ref}}}}}}}$: model-derived phases; m^ref^: figure of merit extracted from the reference data.

The parameter α is inversely related to the occupancy of the triggered state, but its correct value remains unknown until occupancy determination has been carried out. Two methods are available in Xtrapol8 to estimate the triggered state occupancy, either based on the raw extrapolated maps or on the refined extrapolated models. Hence, extrapolated SFAs, maps and models are calculated for each of the user-supplied occupancy values, and occupancy determination is performed at the end of the Xtrapol8 run. By default, and in the *fast-and-furious* mode, only the qF_extr_ type of ESFAs is calculated. Nevertheless, all nine types of ESFAs—or a subset thereof—can be calculated in a single run in the *calm-and-curious* mode, leading to a maximum of 9 independent occupancy estimations by each of the two occupancy determination methods—or 18 estimates.

For each occupancy, extrapolated SFAs and map coefficients are used in three subsequent refinement steps. Firstly, a reciprocal-space refinement of the reference state input model is carried out against the ESFAs, allowing to account for small changes in atomic positions and thereby ameliorate the phases and associated figure of merit *m*, which in turn will result in clearer mF_extrapolated_ − DF_calc_ and 2mF_extrapolated_ − DF_calc_ electron density maps. The latter and the corresponding refined model are then used in automatic real-space refinement, enabling to model conformational changes too large to have been accounted for by reciprocal-space refinement. In addition, real-space refinement of the reference model is also carried out against the initial extrapolated electron density map (calculated before reciprocal-space refinement and update of phases and figure of merits), which offers an unbiased view of the information content in the extrapolated data. The user has the possibility to perform these automatic reciprocal-space and real-space refinement using either phenix.refine^26^ and phenix.real_space_refinement^27^ or refmac^28^ and coot^29^, respectively. An important limitation in streamlining the refinement steps is that they are performed starting from the reference model, which may or may not be adequate to accurately refine the triggered state. With the exception of waters, which can be automatically updated during reciprocal-space refinement when using phenix.refine, no atoms can be removed or added during the Xtrapol8 run. For this reason, it is advisable that automatic refinement is not carried out in studies where covalent bonds are being formed or broken, or if the detection and localization of a ligand is of interest. At the command line, the automatic refinement steps can be disabled by setting the refinement.run_refinement parameter to “False”. When using the graphical user interface XtrapolG8, users need to un-check the “Perform refinement with” option in the *Refinement* panel of the *Configure* tab.

Density modification can aid in meliorating the phase information and cleaning up the electron density maps, offering a better base for real-space refinement. Regardless of the suites of programs used for reciprocal-space and real-space refinement, density modification is performed using the CCP4 program dm^11^. We indeed found that this program is generally unsensitive to the often non-ideal distribution of ESFAs and their standard deviations.

### Negative ESFAs

An often-overlooked issue in structure factor extrapolation is the presence of negative ESFAs. Their origin lies in large negative values of the weighted difference amplitudes (first term in Eq. 2) that cannot be compensated by the additional amplitude term (second term in Eq. 2). For a small occupancy, and thus a large α-value, the percentage of negative ESFAs can become large, which will result in decreasing the true completeness (i.e., the completeness of the positive reflections only) below a critical value of 90%. Indeed, negative SFAs are not handled by refinement programs so that the refinement is carried out against incomplete data, resulting in weak electron density maps and non-converging refinement. In Xtrapol8, we propose different approaches to correct for this issue. In the first approach, the negative reflections are removed, on the ground that the error in the measurements of the associated reflections is too large to lead to a reliable estimate^5^. However, rejecting these may strongly alter the resulting extrapolated maps while biasing the result toward the positive difference amplitudes. In the second approach, the artificial negative reflections are set to zero, under the assumption that true values of these physically-impossible negative ESFAs are weak, but this treatment implies losing important information on their relative strength^14^ and again biasing the result toward the positive amplitudes. In the third approach, the negative ESFAs can be replaced by their corresponding values in either the observed $\left({F}_{{{{{{\rm{obs}}}}}}}^{{{{{{\rm{reference}}}}}}}\right)$ or calculated $\left({F}_{{{{{{\rm{model}}}}}}}^{{{{{{\rm{reference}}}}}}}\right)$ reference datasets, under the assumption that the strong negative difference amplitude is a consequence of measurement errors in ${F}_{{{{{{\rm{obs}}}}}}}^{{{{{{\rm{triggered}}}}}}}$ and that the triggered state closely resembles the reference state. Predictably, however, this approach will bias results towards the reference state, which can lead to an unreliable occupancy estimate and alteration of the extrapolated maps. Importantly, usage of this option should mirror the ESFA-calculation strategy (Table 1), i.e., negative ESFAs should be replaced with ${F}_{{{{{{\rm{obs}}}}}}}^{{{{{{\rm{reference}}}}}}}$ in the case where (*q/k*)F_extr_ and (*q/k)*F_genick_ ESFAs are used, but with ${F}_{{{{{{\rm{model}}}}}}}^{{{{{{\rm{reference}}}}}}}$ when ESFAs are calculated using the (*q/k*)F_extr,calc_ method. Finally, just as with intensities originating from a diffraction experiment with a fully occupied crystal, also the extrapolated intensities, calculated as the square of the ESFAs multiplied by their initial sign, are supposed to follow the Wilson distributions^30^. Structure factors can be re-calculated from the extrapolated intensities by the use of an algorithm taking the French-Wilson algorithm^14^ into account, e.g., truncate^15^. This approach yields the lowest *R*_work_ and *R*_free_ values in reciprocal-space refinement and the most defined electron density around atoms in the model—as judged by higher CC_mask_ and CC_volume_ after real-space refinement—hence it is the default. This option has yet the drawback that all structure factors are being re-calculated, not just the negative ones. Hence, in cases where negative ESFAs amount to less than 5 % of all ESFAs, other strategies may become preferable.

### Occupancy estimation

Determination of the occupancy of the triggered state is one of the main features of Xtrapol8. Indeed, the ESFAs vary as the occupancy changes (Eq. 2), hence incorrect estimation of the occupancy could lead to under- or overrefinement of structural features in the triggered state. Xtrapol8 features two complementary methods for the occupancy estimation, both fully based on the X-ray data without any external assumption or prior knowledge about the triggered state, and with only minimal expectations on the behavior of the structure factors and map coefficients. Both methods can make use of all atoms in the asymmetric unit; of atoms displaying the strongest peaks in the Fourier difference map (as identified by *map-explorer*); or of a user-defined selection of residues, ligands and waters.

The first method, the *difference-map* method, relies on the analysis of the initial extrapolated difference map, i.e., that obtained before refinement of the structure (mF_extrapolated,occ_ − DF_calc_; last column in Table 1). When calculated using the correct reciprocal-space occupancy (α) value, this map strongly resembles the Fourier difference map, with positive and negative peaks pointing to appearing and disappearing features with respect to the reference model, respectively. Peaks in the mF_extrapolated,occ_ − DF_calc_ maps grow in height as the reciprocal occupancy α increases towards the correct value, and then decrease upon further augmentation of α due to an increase in the map noise level. The occupancy that yields the extrapolated difference map with the highest signal-to-noise (S/N) ratio is retained as the most probable one. In practice, we first integrate all peaks in the difference map; use the sum of the integrated absolute values of all peaks as a measure of the noise level in the map (N); utilize a Z-scoring approach to single out the most significant peaks and use the sum of their integrated absolute values as a measure of the signal level in the map (S); plot the normalized S/N ratio as a function of α; and pick as the correct occupancy that for which S/N is maximized.

The *difference-map* method is most closely related to the experimental data. However, it only provides solid results when the Fourier difference map, and thus the extrapolated difference maps, contains features that can be clearly distinguished from noise. In cases where this condition is not fulfilled, the peaks in the Fourier and mF_extrapolated,occ_ − DF_calc_ maps are small, hence the occupancy determination based on the maximization of the S/N ratio may fail. This might happen when multiple species occupy the same site and reduce each other’s signal, or if it was not possible to collect enough data for the reference or triggered state. In such cases, users are advised to check if the default parameters used for the analysis—i.e., maximal and minimal heights for peak selection and integration, and Z-score filtering—are not too stringent for their data, and re-run the analysis using slightly modified parameters.

If occupancy determination by the *difference-map* method nonetheless fails, users can still rely on the *distance-analysis* method, which estimates the occupancy based on the evolution of differences in interatomic distances between the reference model and the models automatically refined at increasing *α* values. Indeed, we found empirically that atoms which undergo positional changes in the triggered state move from their initial position in the reference model to their final position in the extrapolated structures with a sigmoidal dependence—i.e., after a slow increment, the distance difference increases steadily with α and then levels at a plateau value indicating that only noise is being added to the maps upon further increasing α. Thus, an estimate of the α value can be extracted by (1) fitting a sigmoid to the distance changes in function of α for each pair of atoms (*i, j*) (d_(*i, j*)_ = f(α)); (2) extracting α_*i,j*_ as the α-value closest to the point where those shifts are 99% of the plateau; and (3) plotting the distribution of α_*i,j*_ and retaining the average 〈α_*i,j*_〉 and maximum in the distribution (Supplementary Methods). Inasmuch as that the difference signal contributes systematically to the ESFAs (as opposed to noise), the *distance-analysis* method will provide a reliable estimate of the occupancy—even in cases where the difference signal is too weak to visually standout in the Fourier difference map.

### The ‘*fast-and-furious’* and ‘*calm-and-curious*’ modes

In the default ‘*calm-and-curious’* mode, Xtrapol8 performs calculations in the most thorough way possible. For rapid data evaluation (e.g., during data collection) or to estimate the parameters for a next run, calculations can be expedited using the ‘*fast-and-furious’* mode. In this mode, default parameters will be used for most arguments, i.e., only the *q*-weighted Fourier difference map, and ESFAs and map coefficients of the qF_extr_ type will be calculated (Table 1), and refinements will only be performed at the occupancy estimated by the *difference-map* method (Fig. 1, steps 1–5). As a consequence, occupancy determination using the *distance-analysis* method is not possible in the ‘*fast-and-furious’* mode. As most of the Xtrapol8 runtime is occupied by the refinement steps, this mode reduces the overall runtime greatly, allowing crystallographers in the midst of an experiment to take decisions swiftly. Xtrapol8 can also be run in a ‘*Fo-Fo map only*’ mode in cases where users only need to calculate a Fourier difference map (Fig. 1, steps 1-2).

### Output

The output of an Xtrapol8 run is stored in a directory whose name can either be defined by the user or automatically called ‘Xtrapol8’. This directory will contain the Fourier difference map, a log file, a Pymol session, and various figures meant to help in the evaluation of data quality and interpretation of results (Supplementary Fig. 6). The log file contains the most important information, such as statistics on the input files, the location of output files, the number of negative ESFAs and the results of the occupancy estimation. A subdirectory is created for each tested occupancy value, with the name indicative of whether ESFAs were *q*-weighted*, k*-weighted or not weighted. These subdirectories will contain the ESFAs as well as the results from refinements. For each type of ESFAs, a coot session is provided in the subdirectory corresponding to the estimated occupancy.

### Good users’ practice

**Fig. 3:**
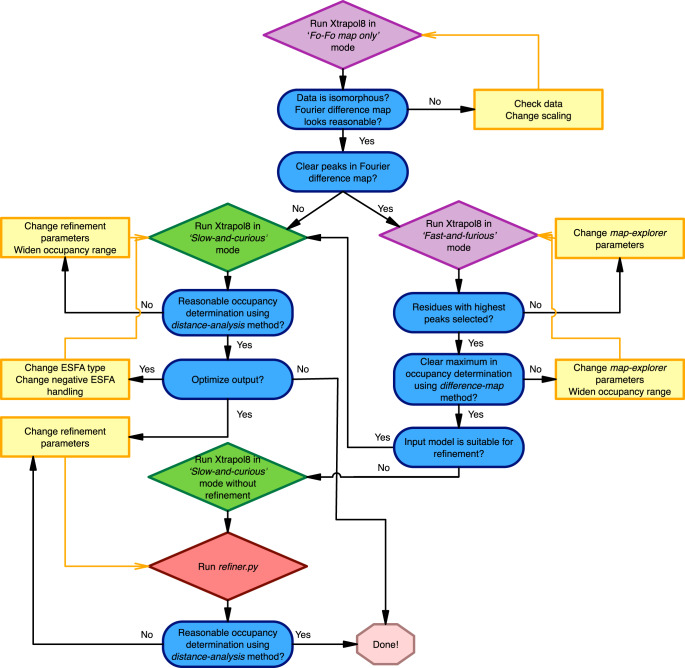
Recommended usage of Xtrapol8. Suggested workflow for efficient usage of Xtrapol8. Users are advised to first run the program in the ‘*Fo-Fo map only*’ mode in order to evaluate the height of peaks in the difference Fourier map. Secondly, it is recommended to run Xtrapol8 in ‘*fast-and-furious*’ mode (purple boxes) to obtain a crude estimate of the occupancy based on the *difference-map* method, and a first characterization of the triggered state structure. Finer exploration can then be carried out using the ‘*calm-and-curious*’ mode (green boxes), which will refine the occupancy determination based on the *difference-map* method, produce refined structures for all tested occupancies and ESFA strategies, and enable orthogonal occupancy determination by the *distance-analysis* method. Evaluation criteria are shown in blue, user actions are depicted in yellow.

The main ambivalence with extrapolation is that in order to calculate the most plausible set of ESFAs, the occupancy must be known. However, occupancy estimations based on X-ray data can only be carried out after the initial extrapolated electron density maps (*difference-map* method) or refined extrapolated models (*distance-analysis* method) have been produced. Therefore, the scheme outlined in Fig. 3 is advised to obtain optimal results in the shortest time. Running Xtrapol8 in the ‘*calm-and-curious*’ mode with improper settings would indeed result in a waste of (computation) time, notably when the program is used to monitor the progress and success of an experiment. Most appropriate is therefore that users run Xtrapol8 in the ‘*Fo-Fo only*’ or ‘*fast-and-furious*’ mode until good parameters are found. A first good sign is that scaling statistics look reasonable, i.e., *R*_iso_ (Eq. 3) and CC_iso_ (Eq. 4) display decent values (*R*_iso_ < 0.20; CC_iso_ > 0.80) even in the highest resolution shell (*R*_iso_ < 0.40; CC_iso_ > 0.60). In the case they are not, users should check their data processing and merging statistics and the unit cell parameters of the reference and triggered datasets in order to increase their chances of getting useful results.

Ideally, the Fourier difference map should cover specific residues (e.g., active sites residues) and the 2mF_extrapolated_ − DF_calc_ electron density maps should be of acceptable quality (i.e., cover all atoms in the model without breaks in the electron density nor spurious peaks in the solvent channels). It may be, however, that no peaks are visible in the Fourier difference maps, in which case users should proceed with extrapolation so as to verify whether or not information is present in the 2mF_extrapolated_ − DF_calc_ and mF_extrapolated_ − DF_calc_ electron density maps. When this is not the case, the scaling parameters and resolution boundaries may need to be altered.

Finding the correct occupancy of the triggered state may require optimization. If features in the Fourier difference map are clear, the default *difference-map* method with preset parameters should provide proper results for the estimated occupancy. A wider range of possible occupancies should be tried, and parameters for the *map-explorer* altered if no clear maximum emerges in the plot of mF_extrapolated,occ_ − DF_calc_ S/N ratios as a function of α. Features in the difference maps may be genuinely weak, in which case the *difference-map* analysis will not allow to discriminate between tested occupancy values, yet the *distance-analysis* method may offer solace. This method however relies on the real-space refined models, and thus requires to run Xtrapol8 in the ‘*calm-and-curious*’ mode. Alternatively, users that have already run Xtrapol8 in the ‘*fast-and-furious*’ mode can use the *refiner.py* script (Supplementary Methods) to refine models and apply the *distance-analysis* method – either starting from the reference model or a manually-modified model. Additional details concerning the optimization of the results can be found in the Supplementary Methods.

### Test case: kinetic crystallography data which allowed identification of a long-lived dark state in mEos4b

**Fig. 4:**
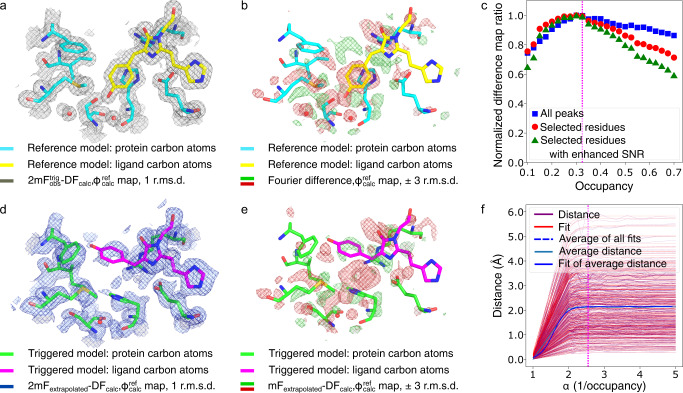
Xtrapol8 enables successful extraction of the mEos4b *red-off* state. The models in cyan and green are mEos4b in the *red-on* and *red-off* state, respectively, as downloaded from the PDB (PDB entry 6GP0 and 6GP1, with the only difference that features of green mEos4b were omitted) with the chromophore indicated in yellow (reference state, **a**, **b**) and magenta (triggered state, **d**–**e**). **a** traditional 2mF_obs_ − DF_calc_ electron density after rigid body refinement of the *red-on* state model in the *red-off* data indicates the absence of signal for the *red-off* state. **b** *q*-weighted Fourier difference map (${F}_{{{{{{\rm{obs}}}}}}}^{{{{{{\rm{red}}}}}}-{{{{{\rm{off}}}}}}}-{F}_{{{{{{\rm{obs}}}}}}}^{{{{{{\rm{red}}}}}}-{{{{{\rm{on}}}}}}}$) superposed on the *red-on* model. **c** the *difference-map* method maximizes the information content of extrapolated difference maps and estimates the occupancy to be 0.325 (magenta dashed line). **d**–**e** Initial *q*-weighted 2mF_extrapolated_ − DF_calc_ (**d**) and mF_extrapolated_ − DF_calc_ (**e**) electron density maps calculated for an occupancy of 0.325 superposed on the *red-off* model. **f** The *distance-analysis* method, which approximates the correct occupancy by fitting a sigmoid to the evolution of interatomic distances as a function of all tested occupancies and then retrieves the occupancy value for which 99% of the plateau is reached, returns an occupancy estimate of 0.380.

The green-to-red photoconversion of mEos4b is the basis of its usage as a marker in photo-activated localization microscopy (PALM). Yet the existence of reversible dark-states, which form upon excitation of the red-emitting state, has long limited its application in single-particle-tracking (spt) PALM. Based on KX experiments, whereby crystalline mEos4b in the green-emitting state was first slowly converted to the red-emitting state and then illuminated by a 561-nm laser before freeze-trapping, a long-lived dark state was identified whose characterization enabled the design of a new data collection scheme suited for the recording of long tracks in spt-PALM^31^. In the reference (*red-on*) and triggered (*red-off*) datasets, two and three states co-exist, respectively, illustrating how complex investigated structural dynamics may be in TRX and KX studies (Fig. 4a).

We first ran Xtrapol8 in the ‘*Fo-Fo map only*’ mode to ascertain both isomorphism of the data and occurrence of conformational changes in the triggered dataset. The isomorphism between the reference (PDB entry 6GP0) and triggered dataset (PDB entry 6GP1) is high, with an overall *R*_iso_ of 0.106 (highest resolution shell *R*_iso_= 0.261; 2.5% increase in unit cell volume; Supplementary Fig. 7a). The *q*-weighted Fourier difference map (${F}_{{{{{{\rm{obs}}}}}}}^{{{{{{\rm{red}}}}}}-{{{{{\rm{off}}}}}}}-{F}_{{{{{{\rm{obs}}}}}}}^{{{{{{\rm{red}}}}}}-{{{{{\rm{on}}}}}}}$) shows strong features on the chromophore and surrounding residues up to a resolution of 1.5 Å (Fig. 4b). Running Xtrapol8 in the ‘*fast-and-furious*’ mode, we tested eight occupancies in the 0.1–0.7 range. Occupancy estimation using the *difference-map* method pointed to the triggered state (*red-off*) displaying an occupancy between 0.3 and 0.4 (Supplementary Fig. 7b). Altogether, Fourier difference map calculation, estimation of the occupancy and production of reciprocal and real-space models of the triggered state took 20 min on a mid-range laptop.

Subsequently, Xtrapol8 was run in the ‘*calm-and-curious*’ mode. By testing 25 occupancies, the final occupancy was determined to be 0.325 (Fig. 4c) using the *difference-map* method. The initial extrapolated mF_extrapolated_-DF_calc_ and 2mF_extrapolated_-DF_calc_ map confirmed the occurrence of large structural changes in the chromophore and surrounding residues (Fig. 4d, e). Some of these could not be modeled automatically during reciprocal and real-space refinements, requiring manual intervention to model chromophore isomerization and accompanying conformational changes. The final triggered state model was characterized by *R*_work_/*R*_free_ and CC_mask_ values of 20.91/23.81% and 91.48%, respectively (compared to values of 15.55/19.00 and 94.67 %, respectively, for mEos4b in the reference *red-on* state)^31^. Similar results were obtained when other types of ESFAs and weighting schemes were used (Table 1; Supplementary Figs. 8, 9). Electron density features were only less pronounced in the case of (*q/k*)Fgenick extrapolated maps^5^ or *k*-weighted extrapolated maps with a high *k*-scale outlier rejection factor. This observation suggests that recalculating figures of merit for each set of ESFAs benefits extraction of structural features for the triggered states, enabling the observation of lower occupancy structural changes. The use of maximum likelihood weighted maps is also likely beneficial, as it allows one to take into account not only errors on phases (m^ref^ or m) but also those on the measurement and estimation of SFAs (D). Specific to mEos4b, similar results were obtained with all possible treatments of negative ESFAs implemented in Xtrapol8, i.e., when they were rejected, set to 0, replaced by ${F}_{{{{{{\rm{obs}}}}}}}^{{{{{{\rm{reference}}}}}}}$ or ${F}_{{{{{{\rm{calc}}}}}}}^{{{{{{\rm{reference}}}}}}}$, or rescued by use of the *truncate* method (Supplementary Figs. 10, 11). This is likely due to their low amount in ESFAs calculated for an occupancy of 0.325 (ranging from 3.0 to 11.2% depending on the ESFA calculation strategy).

The manually-modified triggered state model was finally subjected to automatic reciprocal and real-space refinements against all sets of ESFAs and maps calculated for different occupancies, respectively, enabling the performance of the *distance-analysis* method for occupancy estimation to be verified. To this end we used the *refiner.py* script (Supplementary Methods), which allows the relaunching of all refinements and offers the possibility to run the *distance-analysis* method based on the refined models. The occupancy was thereby estimated to be 0.38 (Fig. 4f), offering orthogonal confirmation for the occupancy determined by the *difference-map* method. The *distance-analysis* method was hardly sensitive to the number of atoms used for the estimation, yielding similar results when either all protein atoms or exclusively atoms with strong difference map peaks were used.

### Other test cases

**Fig. 5:**
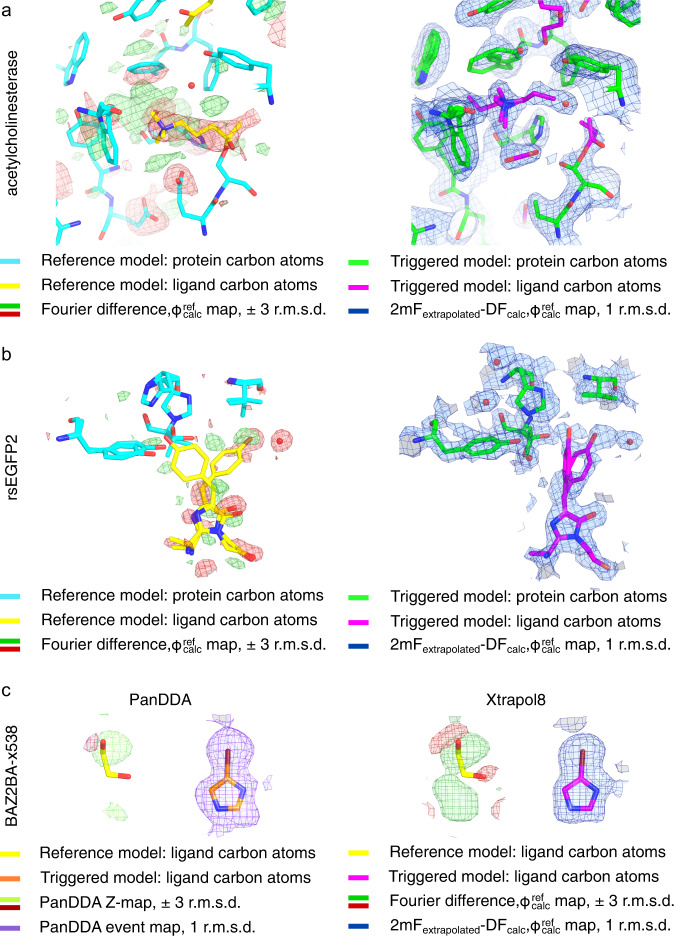
Xtrapol8 enables extraction of low-occupancy states in time-resolved and ligand-based crystallography. In the Supporting results and discussion, we evaluate the versatility and performance of Xtrapol8 by revisiting other time-resolved (TRX), kinetic (KX) and ligand-binding crystallographic studies. For each case the Fourier difference map superposed on the reference state (with carbon atoms of the proteins and ligands colored in cyan and yellow, respectively) and extrapolated electron density map superposed on the triggered state model (with carbon atoms of the proteins and ligands colored in green and magenta, respectively) are shown. **a** A temperature-dependent KX study was conducted on the covalent complex of acetylcholinesterase with a non-hydrolysable substrate analog, whereby X-rays were used to radiolytically cleave bonds, including disulfide bridges and the bond tethering the substrate analog to the catalytic serine^35^. By use of extrapolation, deeper insights could be obtained revealing two binding poses for the radiolytically produced carbocholine product, trapping of CO_2_ from radiolysis of buried acidic residues. **b** A TR-SFX study was conducted on the reversibly fluorescent protein rsEGFP2 with aim to determine the structure of the excited state that preludes isomerization and *off*-to-*on* fluorescence switching^24^. Xtrapol8 allowed obtaining similar results as published earlier for the 1 ps time delay dataset. In the Supplementary results and discussion, we show that Xtrapol8 further allows extending the results obtained at the 3 ps time delay. **c** Comparison of the performance of PanDDA and Xtrapol8 in revealing the electron density of a small compound in a fragment-screening study (BAZ2BA-538)^36,37^.

In the Supplementary Results and Discussion sections, we revisit other TRX, KX and ligand-binding studies that required high-end expertise in crystallography and extensive data processing, yet could be addressed within hours by use of Xtrapol8 (Fig. 5, Supplementary Figs. 12–24). We show in at least two cases (see rsEGFP2 and Shoot-and-Trap test cases) that superior results could have been obtained by the use of Xtrapol8, and how beneficial the proper handling of negative ESFAs can be for modeling and refinement. By enabling automatic elucidation of low-occupancy intermediate states, thereby minimizing the time required to extract meaningful results, Xtrapol8 may accelerate discoveries in TRX, KX and ligand-binding studies. Additionally, it should benefit systematicity and reproducibility of extrapolated map calculation and triggered state structure refinement.

## Conclusion

We here introduced Xtrapol8, a software aimed at standardizing Fourier difference map calculation and application of extrapolation methods. Unique to Xtrapol8 is that it tackles a variety of issues related to Fourier difference maps and extrapolation methods, most notably the need to (i) weight structural observations to maximize the information contents of difference SFAs; (ii) rescue negative ESFAs which can result in sub-optimal refinement and electron density maps; and (iii) determine the triggered state occupancy based solely on X-ray data. As Xtrapol8 offers the possibility to calculate all types of ESFAs at once, and to compare results, it should increase reproducibility while allowing users to make informed decisions as to the method best suited for their project. A level of customization is offered on most important parameters, but defaults are carefully set and Xtrapol8 can be run from the command line or via a GUI, so that adequate results are within reach for experienced and novice users.

## Methods

We applied Xtrapol8 on several sets of published TRX, KX and ligand-binding data. These examples were run using Phenix^12^ v.1.19 and the associated cctbx modules^32^, CCP4^11^ v.7.1 and Coot 0.8.9 or 0.9.4, unless stated differently. Figures were prepared using Pymol 2.5^33^.

### Supplementary methods

Additional methods concerning the *refiner.py* script and details covering data handling and quality assessment, good user’s practice and occupancy determination are provided in the Supplementary Methods section.

### Statistics and reproducibility

Bayesian weighting of difference SFAs and ESFAs (*q/k*-weighting) is performed as described in ref. ^7^ and ref. ^8^. To find the highest peaks in the difference maps (*difference-map* method), a Z-scoring approach is applied to the selected peaks if they follow a normal distribution (*α* = 0.05), otherwise all selected peaks are used for occupancy determination. For the *distance-analysis* occupancy estimation method, only those distances are maintained that are within the 2-6 Å range and to which a logistic function $\frac{L}{1\,+\,{e}^{-k\left(\alpha -{\alpha }_{0}\right)-1}}$ (with L being the maximum value, k the steepness and α_0_ the α -value of the sigmoidal inflection point) can be fitted with an R^2^ of at least 0.95 and a χ^2^ of 0.5, and with boundaries set to allow for an occupancy of the triggered state between 1 and the maximum sampled occupancy.

### Reporting summary

Further information on research design is available in the Nature Research Reporting Summary linked to this article.

## Supplementary information

Supplementary Information

Reporting summary

## Data Availability

The data and models used during the current study were downloaded from the Protein Data Bank for the following entries: 6GP0 (10.2210/pdb6gp0/pdb), 6GP1 (10.2210/pdb6gp1/pdb), 5DTX (10.2210/pdb5dtx/pdb), 5DTY (10.2210/pdb5dty/pdb), 5O8A (10.2210/pdb5o8a/pdb), 2VJA (10.2210/pdb2vja/pdb) and 2VJB (10.2210/pdb2vjb/pdb). The BAZ2BA dataset was downloaded from Zenodo (10.5281/zenodo.48768).

## Code availability

The code and user manual are available at https://github.com/ElkeDeZitter/Xtrapol8, under MIT license^34^. Xtrapol8 makes use of the ccp4^11^ and Phenix^12^ crystallographic suites, as well as the cctbx modules^32^ which are available upon Phenix installation.
