# Supplementary material for: Xtrapol8 enables automatic elucidation of low-occupancy intermediate-states in crystallographic studies
Source: Commun Biol. 2022 Jun 29;5:640. doi: 10.1038/s42003-022-03575-7 (PMC9243118; doi:10.1038/s42003-022-03575-7)
Supplement: Supplementary file 1 — Supplementary Information [file 42003_2022_3575_MOESM1_ESM.pdf]

Xtrapol8 enables automatic elucidation of low-occupancy intermediate-states in crystallographic studies

Elke De Zitter[1], Nicolas Coquelle[2], Paula Oeser[1], Thomas RM Barends[3], Jacques-Philippe Colletier[1*]

[1] Univ. Grenoble Alpes, CEA, CNRS, Institut de Biologie Structurale, 38000 Grenoble, France.

[2] European Synchrotron Radiation Facility (ESRF), BP 220, 38043 Grenoble, France.

[3] Max-Planck-Institut für medizinische Forschung, Jahnstrasse 29, 69120 Heidelberg, Germany.

* Correspondence: colletier@ibs.fr

***

Supplementary information

Supplementary Methods

Data handling and quality assessment: structure factors and intensities

The input data sets can be provided in the form of structure factors or intensities. If both intensities and amplitudes are given, the program will use the amplitudes. In addition to anisotropic and isotropic scaling of the observed data an option is offered to not scale the data at all to account for the possibility that these may have been already scaled– *e.g.,* if merging and scaling of serial time-resolved data was performed using Partialator[1] with the --custom-split option, or if merging and scaling of rotational triggered data was carried out using the reference dataset in XSCALE[2] or Aimless.[3]

Occupancy estimation by the distance-analysis method

By default, the *distance-analysis* method will use atoms to which Fourier difference peaks have been assigned by *map-explorer*, and whose change in distance relative to the reference model is at least 0.25 Å in all extrapolated structures. The program will examine all interatomic distances within the 2-6 Å range (*i.e.,* excluding covalent bond and long-range interactions) that can be fit to the logistic function $^{L}/_{1+e^{-k(\alpha-\alpha_0)-1}}$ (with L being the maximum value (or 1 under normalized conditions), k the steepness and α_0 the α -value of the sigmoidal inflection point) with an R^2 of at least 0.95 and a χ^2 of 0.5, and with boundaries set to allow for an occupancy of the triggered state between 1 and the maximum sampled occupancy. In the case that the user wants to alter the list of residues applied to estimate the occupancy, the stand-alone script *distance_analysis.py* can be used to re-run the distance analysis without having to relaunch Xtrapol8. The user can choose to run the analysis using the list of residues featuring peaks in the Fourier difference map before or after Z-scoring, his own list of residues or no list at all in which case all atoms that are present in all models will be examined. Usage of this script is particularly useful in cases where the *refiner.py* script (see below) was used to launch refinements against ESFA and

extrapolated maps based on a manually modified model. Note that in the ideal case where strong peaks are visible in the Fourier difference map, the *distance-analysis* method can be used as an orthogonal occupancy determination method to confirm the occupancy estimation by the *difference-map* method.

Refiner.py

In the case where the triggered state structure features different atoms as compared to the reference state (ligand based studies, rapid-mixing TRX studies, pump-probe TRX studies with caged-compounds; see the cases of BAZ2BA-x538 and Shoot-and-Trap below), or if the triggered state diverges so much from the reference state that neither reciprocal-space nor real-space refinement can model the structure (*e.g.*, large conformational changes such as fluorescence protein chromophore isomerization; see the case of mEos4b), a better model will become available for refinement of the triggered state upon manual intervention. The latter may then be subjected to reciprocal-space and real-space refinement against all ESFAs pre-calculated for a range of occupancies using the stand-alone *refiner.py* script distributed with Xtrapol8. The script, which at present only works with phenix.refine[4] and phenix.real_space_refine,[5] can take lists of specific arguments for automatic real and reciprocal-space refinement, provided by the user in the form of Phenix input files (we refer to the documentation of Phenix[6] concerning supported input files and parameters). The *refiner.py* script offers the option to recalculate the occupancy by applying the *distance-analysis* method to the refined models. The analysis can be performed using all atoms in the model or a list of residues can be provided. This can be the list written by Xtrapol8 based on the analysis of the Fourier difference map either before or after Z-scoring, or modified by the user, *e.g.,* in cases where only active site or ligand atoms are to be considered for the estimation of the occupancy or if prior knowledge is available as to which atoms undergo significant conformational changes upon triggering of the studied reaction. A compulsory input is the Xtrapol8_out.phil file, written at the end of the preceding Xtrapol8 run, and it is advised to run the *refiner.py* script in the same directory as that used to launch Xtrapol8. Just as for Xtrapol8, *refiner.py* should be launched with phenix.python.

Good users' practice: additional recommendations

Specific to serial crystallography experiments, and provided that difference peaks are visible in the Fourier difference maps, users should continue collection of triggered and reference data until the peak height does not increase anymore as a function of the number of indexed patterns (using the '*Fo-Fo only*' option to stop Xtrapol8 after the generation and analysis of the Fourier difference map). The authors note that they have witnessed cases where rapid calculation of Fourier difference and extrapolated electron density maps were the means by which problems in the experimental setup or crystalline system were diagnosed during TRX experiments. Such cases illustrate the usefulness of having at hand a program like Xtrapol8 in the course of a TRX or KX experiment.

To increase the success rate of the *difference-map* method, users may need to decrease or increase the number of difference peaks used in the *map-explorer* analysis, which can be done by tweaking (1) the maximum (peak_detection_threshold parameter; default is ± 4 r.m.s.d.) and minimum height

(peak_integration_floor parameter; default is ± 3 r.m.s.d.) for peak selection and integration, respectively; (2) the Z-score filter applied to integrated peaks to select the highest ones for occupancy determination (default is 2, corresponding to the ~4.5% highest peaks); and (3) the search radius for assignment of a peak to an atom (default is set to the maximum resolution). A stand-alone script, *differencemap_analysis.py*, is provided to re-run the *difference-map* analysis without the need to relaunch Xtrapol8.

In order to obtain robust results with the *distance-analysis* method, the refinements of the compared models should each converge (*i.e.,* a sufficient number of refinement cycles should be run so that R_{work} and R_{free} values reach a plateau) and refinement parameters should be chosen carefully. Tweaking the refinement parameters can be performed using the refiner.py script, which also integrates the *distance-analysis* method at the end of the refinement steps. Otherwise, the *distance-analysis* can also be carried out using its standalone version, *distance-analysis.py.*

It is evident that not only difference signals but as well noise levels increase upon extrapolation of difference data. An illustration of this can be seen from the shape of the occupancy-determination plot produced by the *difference-map* method, which most often shows a maximum rather than a plateau, and frequently needs human interpretation. It may yet be that the occupancy of the triggered state is low, and that the only means by which the triggered state structure can be revealed is by coping with the high noise level in ESFAs and extrapolated maps. In such cases, refinement parameters have to be carefully validated. It may be advisable to reassess the validity and number of water molecules used in refinement, which Xtrapol8 offers to do using the ordered_solvent option in phenix.refine. By this means, the R_{work}/R_{free} values can be improved by a few percent, which in turn results in better maps. In the case where R_{work}/R_{free} values do not converge during refinement, the user may opt for increasing geometry restraints, at the price however of reducing the capacity of atoms to change position at each cycle, resulting in the need for more refinement cycles. Options to control the weight of the X-ray/geometry terms in the maximum likelihood refinement target are included in Xtrapol8, which directly bind to the relevant options in phenix.refine or refmac5. Specific to the latter, a jelly-body refinement mode exists, access to which is also implemented in Xtrapol8. Another option to cope with high noise levels in extrapolated maps is to use density modification under the reasonable assumption that even in the case of extrapolated data, solvent and protein regions are distinct, the electron density histogram is preserved, and phases can be extended in multiple resolution steps. In the case where density modification is performed, the real-space refinement steps will be carried out using the density-modified reciprocal-space refined maps. In cases where R_{work}/R_{free} are too high to achieve trustable refinement (> 50%), *i.e.,* melioration of phases and figures of merit, users may fully skip reciprocal-space refinement (by setting the number of reciprocal refinement cycles to 0), and solely perform real-space refinement in the initial extrapolated maps. If this strategy is followed and density modification selected, the two real-space models produced by Xtrapol8 are based on the initial and density modified extrapolated maps,

respectively. Otherwise, the two real-space models are based on the initial and refined $2mF_{extrapolated}$-DF_{calc} extrapolated maps. Of note, in the more favorable case where structures can be refined properly and R_{work}/R_{free} values both decrease and converge, common refinement strategies are advised. Hence, all important options in phenix.refine (including simulated annealing, occupancy refinement, individual and grouped B-factor refinement (adp), tls refinement, map-sharpening, etc.) and refmac5 (twinning, overall and isotropic B-factor refinement, tls refinement, map-sharpening, etc.) have been integrated in Xtrapol8. It must be noted that in our tests, refinement using refmac5 did not converge in cases where the triggered state occupancies were lower than ~20%, leading to errors in phases and degradation of the extrapolated maps and models.

Supplementary Results

mEos4b additional results

The structure factors of mEos4b in the *red-on* and *red-off* states were downloaded from the Protein Data Bank (PDB entry: 6GP0 and 6GP1, respectively). The cif file for the chromophore (3-letter code IEY for the red state chromophore) and the altered phenylalanine (3-letter code NFA; a consequence of green-to-red photoconversion) were generated using phenix.elbow. In these data, it is notable that because of incomplete green-to-red photoconversion, both the *red-on* and *red-off* states contain a ~ 50 % contribution from the non-photoconverted green state. This implies that the two data set only differ by the contribution of the *red-off* (triggered) and *red-on* (reference) state. Therefore, we decided for this case study, to simplify the reference input model by removing the green state structural features and setting the red-state features to an occupancy of 1.0 for its usage in Xtrapol8. To evaluate the performance and complementarity between different strategies offered by Xtrapol8, we tested: i) the benefit of Bayesian weighting of difference SFAs on Fourier difference map quality, ii) the accomplishment of the different types of ESFAs (Table 1) in enabling accurate modelling of the triggered state, iii) the pertinence of Bayesian weighting of difference SFAs prior to calculation of ESFAs, and iv) the opportune use of our different approaches to handle negative ESFAs.

Xtrapol8 enables calculation of three types of Fourier difference maps, which differs in the weighting scheme applied to difference SFA, *i.e.,* no weighting, *q*-weighting or *k*-weighting. Specific to the latter, we further evaluated the effect of varying the additional user-modifiable scaling parameter to downweigh outliers during *k*-weighting. Indeed, in the literature, values of 0.05 to 1.0 have been used.[7,8] We found that in the mEos4b case, the *q*-weighted map and the *k*-weighted maps calculated with a *k*-weight scale factor of 0 - 0.3 are those featuring the highest quality, as judged from the height of peaks revealing presence and disappearance of the *red-off* and *red-on* states, respectively, and from the reduced number of spurious peaks attributable to noise (Supplementary Fig. 8).

We next challenged the different types of ESFAs in terms of the quality of the $2mF_{extrapolated}$-DF_{calc} electron density map. Examination of extrapolated maps calculated at with an occupancy of 0.325

(Supplementary Fig. 9) point to damped electron-density features for the triggered (*red-off*) state in $2mF_{extrapolated}$-DF_{calc} maps calculated using the (*q/k*)Fgenick type of ESFA [9,10] and, accordingly, electron density remains fully visible for the initial reference (*red-on*) state. Remarkably, a similar trend is observed upon increasing the *k*-weighting outlier scale factor, which suggest that the result will tend towards the reference state if large structure factor differences are rejected from the map-calculation. It should be noticed that while informative Fourier difference maps can be calculated up to a *k*-weight scale factor of 0.3, the bias towards the reference manifests itself as of a *k*-weight scale factor of 0.1 – *i.e.*, only *k*-weight scale factors as low as 0 or 0.05 appear acceptable for the calculation of ESFAs in this case. As noted by the authors in the initial publication, the chromophore in the *red-off* state most probably contains multiple conformations to which a main population can be modeled. This can explain why CCmask values lie in the 0.5 – 0.6 range and the *red-off* chromophore not being entirely covered by clear electron density. Specific to the usability of the various types of ESFAs to estimate the occupancy based on the *difference-map* analysis, we found the predicted occupancy to lie between 0.3 and 0.4 in most cases.

Last we tested the effect on extrapolated map quality and triggered state structure refinement of the various handling schemes implemented in Xtrapol8 to cope with negative ESFAs. For this comparison, we reasoned that the handling of negative ESFAs should mirror that of missing reflections for the calculation of $2mF_{extrapolated}$-DF_{calc} electron density maps, as taken care of by mmtbx.map_tools.[11] Thus, automatic filling of missing map coefficients of the $2mF_{extrapolated}$-DF_{calc} map was performed when the truncate-, $F_{obs}^{reference}$-, $F_{calc}^{reference}$- and zero-strategies were used, but not when the reject-strategy was applied. In the specific case of mEos4b, with a low fraction of negative ESFAs (2.5 – 10.2 % depending on the ESFA type), the weighting scheme and type of extrapolation appears to have a higher influence on the extrapolated electron density and refinement R-values than the negative ESFA strategy (Supplementary Fig. 9, 10 and 11).

Identification of ps-lived excited-states in a reversibly switchable fluorescent protein (rsEGFP2) by use of time-resolved serial femtosecond crystallography

rsEGFP2 is a reversibly switchable fluorescent protein that can be toggled back and forth between a fluorescent *on*-state and a non-fluorescent *off*-state by subsequent illumination with cyan and ultra-violet light, respectively. Just like in mEos4b, the chromophore is a conjugated system consisting of an aromatic phenol ring and an imidazolinone ring that are maintained coplanar by a methylene bridge. The chromophore features a *cis* anionic configuration in the fluorescent *on*-state, but is *trans* protonated in the non-fluorescent *off*-state. Structures of these states have been solved by rotation-based cryo-crystallography at 100 K (PDB entries 5DTX and 5DTY for the *on*- and *off*-state structures, respectively)[12] and by serial crystallography at room-temperature (PDB entries 5O89 and 5O8A for the *on*- and *off*-state structures, respectively).[13] Long debated has been whether the first step of

photoswitching is isomerisation or protonation, and the question was addressed experimentally by performing time-resolved serial-femtosecond crystallography (TR-SFX) at an XFEL[13] on a slurry of rsEGFP2 microcrystals. *Off*-to-*on* photoswitching was followed after pulsed laser illumination at 400 nm to pump the system to the *on*-state. The crystalline system was probed at time delays Δt of 1 and 3 ps after the 400-nm light trigger, with aim to shed light on the structure and decay of the excited state. As a reference, data was also collected on untriggered crystals (*i.e.,* crystals in the initial *off*-state), enabling calculation of a Fourier difference map for the two-time delays ($F_{obs}^{laser\text{-}ON\text{-}1ps} - F_{obs}^{laser\text{-}OFF}$ and $F_{obs}^{laser\text{-}ON\text{-}3ps} - F_{obs}^{laser\text{-}OFF}$). For the 1 ps time delay, ESFAs were calculated and the occupancy of the triggered state was determined to be 7.5 %, based on a forerunner of the *difference-map* method applied to manually-selected residues. This state was found to consist of two conformers; a twisted intermediate (referred to as model T in Ref[13]), with a quasi-perpendicular positioning of the phenol and imidazolinone groups in a so-called 'twisted' chromophore; and a *trans*-like intermediate, with the ring moieties very close to those of the *trans* protonated *off*-state (referred to as model P in in Ref[13]). Modelling of these intermediates was complicated by the comparatively poor quality of the extrapolated maps, due to low occupancy of the triggered state and to a high amount of negative ESFAs, which translated to a reduced completeness of the data used in refinement and map calculations. For these reasons, the authors then refrained from calculating ESFAs from the data collected at Δt=3 ps, which were of lower quality than those collected at the Δt=1 ps time delay. Hence, these pump-probe TRX data collected on rsEGFP2 appeared to us as an obvious test case for Xtrapol8.

The deposited structure features two full chains, present as alternate conformers, reflecting the mixture of *on*- and *off*-states (10 and 90 %, respectively) present in rsEGFP2 crystals upon pre-illumination at 488 nm (PDB entry 5O8A). To simplify the model used for phase calculation, and avoid possible bias from the reference structure, we performed a novel refinement of the *off*-state structure (laser-OFF dataset, 1.7 Å resolution) whereby instead of a full-length *on*-state conformer at an occupancy of 10%, we here accounted for the residual *on*-state by addition of alternate conformers only at loci featuring peaks in the mF_{obs}-DF_{calc} maps (Supplementary Fig. 12 a). The R_{work}/R_{free} values of the two reference models are similar, viz 13.4 / 17.5 % and 14.7 / 17.6 % for the re-refined and deposited models, respectively. Using the laser-OFF (reference) and laser-ON-1ps (triggered) datasets, and the new reference model, we ran Xtrapol8 in the '*Fo-Fo map only*' mode to generate *q*-weighted and *k*-weighted Fourier difference maps, and then in the '*calm-and-curious*' mode to compute unweighted, *q*-weighted and *k*-weighted ESFA for ten evenly-spaced occupancies between 0.05 and 0.7 (Supplementary Fig. 13). Initial occupancy of the triggered state was determined using the *difference-map* method, and amounted to 0.075 - 0.10, depending on whether *q*-weighting (0.075), *k*-weighing (0.10) or no weighting (0.10) was used. In the *'calm-and-curious'* mode, automatic refinement was performed at all occupancies. We found *k*-weighting of ESFAs to be superior to *q*-weighting, yielding 2 - 8 % improvement in initial R_{free} depending on occupancy. With *k*-weighting, and regardless of the occupancy at which extrapolation is

carried out, the standard deviation on the ESFAs (σ(kFextr)) hardly varies with resolution (Supplementary Fig. 13c). Contrastingly, the standard deviations on unweighted (σ(Fextr)) and *q*-weighted (σ(qFextr)) ESFAs show a similar trend, being surprisingly high at low resolution, minimal around 2.5 Å, and rising again at high resolution. Probably, this difference in the determination of σ(ESFA) is at the root of the higher R_{work} and R_{free} values observed for the unweighted and *q*-weighted extrapolated structures, as compared to their *k*-weighted counterparts. The plots showing the weighting factor in function of resolution (Supplementary Fig. 13d) reveal that lower *k*-weights are at the origin of the reduced σ(ESFA) on k-weighted ESFAs at low and high resolution. Thus, *k*-weighting is useful to account for measurements errors at low resolution, here due to the hollow nature of XFEL detector, and at high resolution, due to reduced diffracted intensities. In the refined extrapolated maps, density was visible for the P and T states in the $2mF_{extrapolated}$-DF_{calc} map, regardless of the method to treat negative ESFAs and weighting (Supplementary Fig. 14), and they could be modelled manually in coot. The density for model T is best defined in the *q*-weighted maps, despite an overall lower CC_{mask} compared to *k*-weighted maps; possibly, this is due to a damping of structural differences in the *k*-weighted ESFA, which results in reference state features being more present (and inversely for the triggered state features) in the corresponding maps, thus resulting in a better-defined electron density and higher CC_{mask}. Focusing on the *q*-weighted maps, the extrapolated map calculated from ESFA generated using the truncate method displays the best overall quality, as judged from CC_{mask}. After inspection of the rest of the protein structure and of waters for the structure calculated using *k*-weighted ESFAs at an occupancy of 0.1, the resulting model was subjected to automatic reciprocal-space and real-space refinement against all extrapolated datasets and maps using the *refiner.py* script, offering a view of how conformation and occupancy of the two intermediate states are influenced by the extrapolation occupancy (Supplementary Fig. 12, 15 and 16), and affording to obtain another estimate of the correct value of the occupancy by the *distance-analysis* method (Supplementary Fig. 17). The distance-refinement method fairly reproduces the occupancy determination from the *difference-map* method, regardless of whether *q*-weighted or *k*-weighted or unweighted extrapolated maps are used for real-space fitting, or of whether all atoms or only atoms with $F_{obs}^{laser\text{-}ON} - F_{obs}^{laser\text{-}OFF}$ peaks or only atoms with the highest $F_{obs}^{laser\text{-}ON} - F_{obs}^{laser\text{-}OFF}$ peaks are considered for the estimation. Hence, the *distance-analysis* method can be used to judge extrapolation results and determine the triggered state occupancy even in cases where $F_{obs}^{laser\text{-}ON} - F_{obs}^{laser\text{-}OFF}$ peaks are weak or absent. This may be the case when conformational changes are small in amplitudes or when the triggered state consists (as is the case here) of a mixture of close conformers whose positive and negative density peaks $F_{obs}^{laser\text{-}ON} - F_{obs}^{laser\text{-}OFF}$ overlay resulting in a reduction of the observed difference electron density for each. When all atoms are used, hints can be obtained regarding the presence of different intermediate states present at different occupancies in the triggered state dataset, *e.g.,* the P and T models. Refinement results obtained using the *q*-weighted and

k-weighted ESFA were similar, and point to an occupancy of 10-15%, in reasonable agreement with the *difference-map* method (Supplementary Fig. 16).

Encouraged by these results, we asked whether or not refinement could also be performed against ESFAs calculated for the 3 ps time delay. At the time of publication, the authors refrained from carrying out refinement against ESFAs because of the large amount of negative ESFAs (~20% at 20% occupancy) that resulted in high R_{work} and R_{free} values. We ran Xtrapol8 in the *'calm-and-curious'* mode with ten evenly-spaced occupancies between 0.05 and 0.7. We used the *difference-map* method to estimate the occupancy of the triggered state(s), which returned values of 0.05, 0.075 and 0.1 when *k*-weighting, *q*-weighting or no weighting was used, respectively (Supplementary Fig. 18). Reciprocal-space and real-space refinement were carried out using the same protocol as above. From the extrapolated *k*-weighted $2mF_{extrapolated}$-DF_{calc} map, we were able to model three chromophore conformers: (i) a pseudo-*trans* conformer (closer to the *off*-state *trans* conformer than to the model P observed at Δt=1 ps); (ii) a pseudo-*cis* conformer (close to the *on*-state *cis* conformer but with slightly different dihedral angles); and (iii) a conformer at mid-way between the two latter, featuring nearly the same phi angle as model T but a tau angle similar to the *cis* conformer, and which we therefore coin the anti-twist conformer (Supplementary Fig. 12, 19 and 21). The three-conformer model was refined against all sets of EFSA using the *refiner.py* script again pointing to an occupancy of 5 – 10 %, in line with the prediction of the *difference-map* method (Supplementary Fig. 20). It is noteworthy that similar conclusions can be drawn from the use of *q*-weighted ESFAs and $2mF_{extrapolated}$-DF_{calc} map (Supplementary Fig. 12, 19, 20 and 21), notwithstanding a change in refined occupancies of the for the three conformers.

Xtrapol8 performance in ligand binding and comparison with PanDDA

Fourier difference maps and structure factor extrapolation can also prove useful in cases where the complex structure of a protein with a poorly-occupied ligand is of interest. Indeed, in such cases as well, low occupancy translates to poorly-defined electron density making identification of the binding pose challenging if not impossible. To date, the best solution for such cases is the real-space subtraction and map-deconvolution approach implemented in the software PanDDA,[14] whereby a reference electron density map, averaged from hundreds of ligand-free datasets, is for each ligand subtracted from the electron density map obtained after automatic refinement. To compare the performance of Xtrapol8 and PanDDA in their usability for fragment screening, we selected two datasets, BAZ2BA-x645 (reference) and BAZ2BA-x538 (triggered), from the BAZ2BA fragment screening dataset (downloaded from Zenodo[15]) that was used to showcase the performance of PanDDA in its original publication.[14] Interestingly, the BAZ2BA-x645 dataset was also collected from a crystal soaked with a ligand, but as the latter did not bound, the dataset was automatically identified as a reference dataset by PanDDA. In a regular Xtrapol8 experiment, the reference data set would ideally emanate from an unsoaked crystal.

Whereas only 2 data sets from the BAZ2BA set were used to run Xtrapol8, we ran PanDDA,[14] as part of ccp4-7.0, on the complete set, consisting in hundreds of datasets, without defining a resolution cutoff.

We first ran Xtrapol8 in the '*Fo-Fo only*' mode. The overall R_{iso} value between the BAZ2BA-x645 and BAZ2BA-x538 datasets is 10.2 % over the full resolution range (28.9 - 1.76 Å), but raises steeply to 30.6 % in the highest resolution shell. Therefore, data were cut to 1.85 Å, leading to an overall R_{iso} of 9.8 % and high resolution R_{iso} of 23.9 % (Supplementary Fig. 22a). Xtrapol8 was subsequently run in the '*fast-and-furious*' mode, testing 10 occupancy values between 0.05 and 0.5. As in ligand-binding studies the reference model does not contain the modelled ligand, it is advisable that no refinement steps are run after extrapolation. The *difference-map* method pointed to an occupancy between 0.20 to 0.30. Xtrapol8 was thereafter rerun in the *'calm-and-curious'* mode, using the truncate method to handle the negative ESFAs. Ten evenly-space occupancy values between 0.15 and 0.33 were tested, each using various strategies for weighting and ESFA-calculation. Because refinement was not carried out, occupancy estimation could only be performed automatically using the *difference-map* method. With ESFAs calculated using the *q*Fextr method, we found an occupancy of 0.25 for the 4-bromoimidazole ligand-bound state (Supplementary Fig. 22b). Similar occupancies were predicted when other types of ESFAs were used, ranging from 0.17 (*k*Fextr_calc) to 0.27 (*k*Fgenick), or when PanDDA was used (0.24). The extrapolated *q*-weighted $2mF_{extrapolated}$-DF_{calc} electron density map produced by Xtrapol8 is similar to the event-map calculated by PanDDA for the brominated ligand (Supplementary Fig. 22c and d). Yet, the Fourier difference map of Xtrapol8 surpasses the z-map of PanDDA in that not only the bromide, but as well the imidazole moiety, are visible at a level of 4 r.m.s.d. (Supplementary Fig. 22e and f). Fourier difference peaks could also be found on the protein (Supplementary Fig. 22g and h), pointing to the conformational changes undergone by the protein upon binding of the ligand.

The BAZ2BA test-case provide a good example for the use of Xtrapol8 in structural enzymology where, alike in this example, the reference and triggered state may contain different ligands. This may also apply in the case of time-resolved experiments on crystalline proteins complexed with caged-compounds in which the compound breaks in multiple moieties upon reaction triggering and might not bind to the protein prior to uncaging. In such cases, it may only become useful to perform occupancy determination using the *distance-analysis* method after the triggered structure has been (manually) refined using an occupancy estimate from the *difference-map* method (best case scenario) or decided upon based on the visual inspection of extrapolated maps. A user could then decide to use the *refiner.py* script to refine the manually-fitted triggered-state model against all sets of ESFAs calculated for the various occupancies. This would then open the door to orthogonal occupancy determination by the *distance-analysis* method.

Shoot-and-Trap study of a complex of acetylcholinesterase with a non-hydrolysable substrate analogue

By enabling the rapid hydrolysis of the neurotransmitter acetylcholine into acetate and choline, acetylcholinesterase (AChE) terminates impulse transmission at cholinergic synapses, restoring neuronal excitability.[16] Such synapses are present in the central nervous system, where they support cognitive functions, as well as in the parasympathetic branch of the peripheral autonomous nervous system.[16] Therefore the enzyme is essential, as well as the requirement that it functions at a rate that is nearly diffusion-limited, *i.e.,* ~1000-10000 s^{-1} depending on species. Because the active site of AChE is buried at the bottom of a ~20 Å deep aromatic gorge,[17] the molecular basis for the high turnover rate long remained unclear, and likewise for the mechanism underlying substrate inhibition at high concentrations.[18] It was proposed based on early molecular dynamics simulations that the choline product could exit through a backdoor,[19] which would transiently open at the bottom of the gorge, where the enzyme wall is the thinnest, following rupture of H-bonds between the polar side chain atoms of W84, W432 and Y442. Thereby, substrate and product traffic in the active site gorge would be alleviated, contributing to the high turnover rate. In 2006, structures of AChE in complex with isosteric hydrolysable and non-hydrolysable substrate analogues were produced, revealing the molecular basis for substrate binding and inhibition.[20,21] The non-hydrolysable substrate analogue (4-oxo-N,N,N-trimethylpen-tanaminium; OTMA) can bind both at the active and the peripheral site, located at the bottom and entrance of the gorge, respectively, leading to closure of the gorge at mid height. In the active site, the analogue covalently binds to catalytic Ser200 from the catalytic triad, with the H-bonding pattern between the catalytic triad residues (E327, H440 and S200) remaining unaffected. However, the electron density of the analogue was reduced around the C5-C6 bond, which replaces the hydrolysable C-O bond of the natural substrate, illustrating the electron-withdrawing effect of covalent binding to catalytic S200. It is this observation that led to a subsequent study on this complex, whereby data was collected before and after rupture of the substrate analogue C5-C6 bond by prolonged exposure to X-rays, in an approach coined "Shoot-and-Trap".[22] By means of q-weighted Fourier difference map calculations, it was shown that the carbocholine radiolysis product reorients in the active site following radiolytic cleavage of the substrate analogue at 100 K, but escapes the active site at 150K. A succession of positive and negative peaks in the map were observed on the residues proposed to be involved in the opening of a putative backdoor, offering first structural support for its existence.[19] Disulfide bond disruption upon exposure to X-rays was also evident from the q-weighted Fourier difference maps at both temperatures. Additionally, evidence was obtained for the trapping two CO_2 molecules produced by radiation-induced decarboxylation of buried acidic residues at 100K.

At the time, however, structure factor extrapolation and reciprocal-space refinement were not attempted by the authors; hence we wondered if further insights could have been obtained by use of extrapolation methods. We therefore subjected the 100 K reference (~3 MGy, *i.e.,* 1/10th of the experimentally

determined Garman limit[23]) and triggered (~9 MGy, *i.e.,* 1/3rd of the Garman limit[23]) datasets to Xtrapol8. In the probed crystalline system, two monomers are found in the asymmetric unit (chains A and B) forming a functional dimer assembled by a four-helix bundle. Below, results and figures refer to monomer A; the results for monomer B are similar unless stated otherwise.

Reference (PDB entry 2VJA corresponding to AChE in complex with the substrate analogue collected with a dose of 3 MGy at 100) and triggered (PDB entries 2VJB corresponding to AChE in complex with the OTMA substrate analog collected with a dose of 9 MGy at 100) datasets were scaled anisotropically before the calculation of a $F_{obs}^{2VJB} - F_{obs}^{2VJA}$ (*i.e.*, $F_{obs}^{9MGy} - F_{obs}^{3MGy}$) *q*-weighted Fourier difference map and corresponding ESFAs for nine evenly-spaced occupancies between 0.1 and 0.9. Occupancies of the triggered states were determined using the *difference-map* method, and amounted to 0.2 (Supplementary Fig. 23). Again, we used the default truncate method to rescue negative ESFAs. As for the BAZ2BA-x538 test-case, carrying-out automatic refinement with the reference model was inappropriate since the reference and triggered states do not feature the same ligands in the active site gorge (substrate-analogue vs. radiolytic products). The $F_{obs}^{2VJB} - F_{obs}^{2VJA}$ *q*-weighted map calculated by Xtrapol8 is as featureful as that reported earlier, which was calculated in CNS using the original *q*-weighting script written by Thomas Ursby[24] (Supplementary Fig. 24).

Structure factor extrapolation allowed us to expand on the insights obtained in the initial study. Indeed, the $2mF_{extrapolated}$-DF_{calc} and $mF_{extrapolated}$-DF_{calc} maps reveal the presence of four radiolytic products in the active site: (i-ii) carbocholine and ethanal, from the radiation induced cleavage of the C5-C6 bond in the substrate analogue; and (iii-iv) two CO_2 molecules, from the decarboxylation of E199 and catalytic E327. Several residues in the active site are present in alternate conformations. Notably, two conformations are seen for carbocholine. In both, the choline moiety interacts by cation-π interactions with W84 and F330, yet each conformer coincides with different F330 conformations and different water networks. The first carbocholine conformer has its carbon chain pointing towards catalytic S200, while that of the second conformer points towards W432 and Y442, following a 180° rotation around the choline nitrogen position. Importantly, neither of the two carbocholine binding modes corresponds to that reported earlier for thiocholine [20] and choline,[21] either due to the change in their terminal atom (C, S, O, respectively) or to the void left by the decarboxylation of E199. Two close conformations are as well observed for the ethanal product, which is best modelled as either bound covalently to the catalytic S200 (conformer A, with the ethanal oxygen atom H-bonding (2.7 Å) to A201(N) in the oxyanion hole) or at Van der Waals distances from its side chain (conformer B, with the ethanal oxygen atom pointing towards S226(O)). In both cases, the methyl group faces the acyl-biding pocket, but only in conformer B are the catalytic S200 and H440 H-bonded, despite the latter adopting a conformation different from the resting state on the two alternate conformations. Indeed, a ~60° change in H440 side chain conformation is observed, presumably as a result from the untethering from E327, due to

decarboxylation of this residue and repositioning of the CO_2 in the vicinity of S226(OH). The distribution of alternate conformations allows a global picture to emerge, whereby the carbocholine with its carbon chain pointing towards S200, the covalently bound ethanal, the gorge-closing F330 conformer and the CO_2 molecule emanating from E199 decarboxylation coexist in a first conformer (conformer A), whereas after CO_2 exit, the carbocholine reorients, the ethanal is released from S200, F330 assumes its gorge-opening conformation and a change in the conformation of S200 restores H-bonding to H440 (conformer B). The first set of alternate conformers can be proposed as representative of the structure just after cleavage of the substrate analogue, whereas the second set could correspond to the subsequent step. At the peripheral site, decarboxylation of a glutamate is also observed (E278), with the radiolytically-produced CO_2 molecule remaining trapped in a groove lined by the side chains of W279 and Y70, and the main chain carbonyl and side chain of I275. This causes W279 and Y70 to adapt a double conformation, which might be the reason for the absence of the substrate analogue at the peripheral site. Rather, a double conformation PEG molecule can be fitted, which adopts different conformations in the two monomers of the asymmetric unit.

As suggested by the $F_{obs}^{2VJB} - F_{obs}^{2VJA}$ map, radiation-induced decarboxylation is most evident at the functionally important E199, E327 and E278. Refinement based on extrapolated maps nonetheless indicates that this process occurs throughout the protein. Indeed, in monomer A (monomer B), only 6 (9) aspartate residues over 24 (24), and 6 (10) glutamate residues over 33 (34; residues 486-489 are undefined in chain A) preserve their carboxylic groups. No clear pattern emerges in terms of the predicted pKa (calculated using propka in pdb2pqr[25]) or burying into the structure of these radiation resistant acidic residues (Supplementary Fig. 23e). Nonetheless, we note that none of the glutamic acid residues preserving their carboxylic group (16 in total) and only two aspartic acid residues preserving their carboxylic group (15 in total) are involved in Coulombic interactions, suggesting a relation between the resistance to radiation damage of acidic residues and the absence of Coulombic interactions involving their side chains. CO_2 molecules could be fitted at 10 (7) loci in monomer A (monomer B), but only three of these are buried in the protein moiety, nearby E199, E278 and E327. It must also be noted that all disulfide bridges (three per monomer: C67-C94, C254-C265, C402-C521) are ruptured in the extrapolated structures, as clearly pointed to by the $F_{obs}^{2VJB} - F_{obs}^{2VJA}$ Fourier difference map and in agreement with Ref[27] (Supplementary Fig. 24b, c, e and f). Surprisingly, the C254-C265 disulfide break could not be unambiguously modeled as two individual cysteines as no density for a sulfur atom at position 254 could be observed. The elongated electron density at position 265 could suggest an asymmetric breaking mechanism at the C254(Cβ)-C254(Sγ) bond, *i.e.*, not at the C254(Sγ)- C265(Sγ) bond (Supplementary Fig. 24b and e), and remains to be further investigated.

The Shoot-and-Trap example offers illustration that more information can be extracted from refinement against ESFAs than from the analysis of a Fourier difference map alone. From the latter, it was rightfully concluded that exposure to high X-ray doses leads to i) breakage of disulfide bridges; ii) decarboxylation

of E199, E278 and catalytic E327; and (iii) cleavage of the non-hydrolysable substrate, with (iv) repositioning of the radio-produced choline adduct at 100 K. Upon structure refinement in ESFAs, we show that the non-hydrolysable substrate analogue is not only cleaved, but that this cleavage occurs at the C5-C6 bond, resulting in the production of carbocholine and ethanal. The carbocholine reorients and adopts two as yet unobserved conformations in the active site, likely due to trapping of CO_2 at the bottom of the gorge, following decarboxylation of E199. One of these shows the carbon chain pointing towards the catalytic S200, and may represent the structure of the protein just after photocleavage of the substrate analogue. The other conformer has its carbon chain pointing towards Y442, following a 180° rotation around the choline position. The two conformers coincide with different conformations of F330 and different water networks. Two close conformations are as well observed for the ethanal product, which is either bound covalently to the catalytic S200 (conformer A) or found at Van der Waals distances from its side chain (conformer B).

Supplementary Discussion

Here we reported the design and use of Xtrapol8, a program aimed at facilitating the determination of low-occupancy state structures in time-resolved, kinetic and ligand-binding crystallography. Examples presented above demonstrate that Xtrapol8 can be useful in a wide variety of cases, from ligand-binding to kinetic and time-resolved crystallography.

Xtrapol8 was designed with view to address three main issues. Firstly, we wanted to make available to a wide community of crystallographers a tool enabling eased calculation of Bayesian-statistics weighted Fourier difference maps and ESFAs, and this independent of their crystallographic expertise. To this end, our software had to be user-friendly and highly-automated, requiring minimal input from inexperienced users while allowing expert crystallographers to tweak the calculations with their preferences. Hence, many parameters such as resolution cutoffs, scaling parameters, weighting schemes, as well as parameters for difference map exploration or refinement can be defaulted or adjusted. To further ease its usage, Xtrapol8 can be controlled through an intuitive graphical user interface as a frontend to the command line version, which we hope will reduce the barriers for inexperienced crystallographers to use extrapolation methods. Secondly, as different approaches for the calculation of Fourier difference map and extrapolation of structure factor amplitudes have been proposed, we sought to make all of them available within a single tool, so as to allow users to compare results obtained using different strategies. Indeed, we observed that depending on the case, a specific strategy might be more successful than another; Xtrapol8 allows testing all methods within a single run. Thirdly, we aspired to address the issue of negative ESFAs, whose percentage increases as the occupancy of the intermediate state decreases, degrading the quality of extrapolated maps while making reciprocal-space and real-space refinement unstable. Presence of negative ESFAs can be unnoticed when using custom-written scripts, leaving their treatment – most often their elimination – to the refinement software that is used downstream. As shown by our examples, the best approach depends on the actual data, the total number of negatives and if any bias can be afforded. If the negative reflections are simply rejected, the effective completeness of the data used in refinement can strongly decrease, leading to a degradation in map quality and refinement results. Last, with view to increase overall reproducibility and transparency, we wanted to provide methods to objectively estimate the occupancy of the triggered state(s) based exclusively on the diffraction data. Two such methods are accordingly available within Xtrapol8. The *difference-map* method maximizes peak height in the extrapolated difference maps, whereas the *distance-analysis* method exploits changes in atomic positions in the triggered state models produced by reciprocal- and real-space refinement against ESFAs and extrapolated electron density maps, respectively, with respect to the reference model. Ideally, both occupancy estimation methods should be used to enable cross-validation of the occupancy estimation. However, usage of the latter method requires proper refinement settings and correct modelling of the extrapolated density map. In some cases, illustrated by our examples, the automatic reciprocal-space and real-space refinement should not

/ cannot be carried out before manual intervention on the model, hence only the *difference-map* method will initially be usable. Nevertheless, a script is provided to re-run the *distance-analysis* method in stand-alone mode so that the user may intervene manually to produce a first model of the triggered state with which the refinements can be relaunched and occupancy determined via the *distance-analysis* method. Importantly, the *distance-analysis* method may be the only valid option in cases where atomic motions are small, data are weak, or multiples excited states coexists, leading to a flattening of peaks in the Fourier difference map.

Of important note, Xtrapol8 can also be used for the evaluation of ligand binding and fragment screening assays, complementing already existing pipelines such as PanDDA.[14] The latter decides on the presence of a potential ligand based on the electron density distribution and then uses an ensemble of ligand-free electron density maps to locate the ligand through real-space electron density deconvolution. In contrast, Xtrapol8 uses a single *a priori* ligand-free data set and bases all occupancy-estimations and structure-refinements on SFA differences. The two methods are thus highly complementary. Indeed, the PanDDA strategy is less dependent on crystallographic isomorphism and generates nearly noise-free local electron density maps, but the calculation does not allow fast feedback and requires an ensemble-based refinement in the initial untreated data. Contrastingly, Xtrapol8 allows to calculate a Fourier difference map in a matter of minutes, allowing real-time feedback, and the ESFAs can be used to refine the structure using standard protocols.

Our examples illustrate how important occupancy estimation can be for the interpretation of structural results. In the case of crystallography experiments where the completeness and multiplicity of the data is high (*e.g.,* serial crystallography), jackknife resampling could be implemented[26] offering a means to increase the statistical significance of the occupancy estimate. To do so, multiple random (reference and triggered) datasets would have to be produced, each lacking a given fraction of the collected frames, enabling to run Xtrapol8 multiple times on slightly different data, and to base occupancy determination on the distribution of the estimated occupancy for each pair of jackknifed datasets. While jackknife resampling is not part of the current Xtrapol8 release, it will be incorporated soon.

Supplementary Figures and Tables

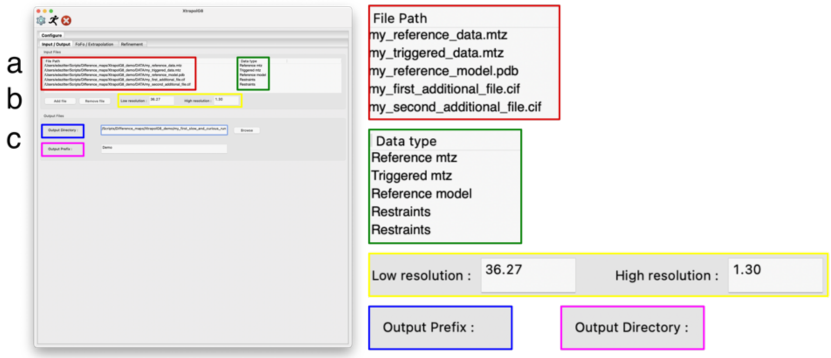

Supplementary Fig. 1 | Panel of the Xtrapol8 graphical user interface for input and output management. Colored boxes indicate zoom-in at several fields. **a,** input files and specification of their type (reference mtz, triggered mtz, reference model and additional cif files if required). **b,** resolution limits can optionally be adapted. **c,** fields to alter the output directory and prefix for output files.

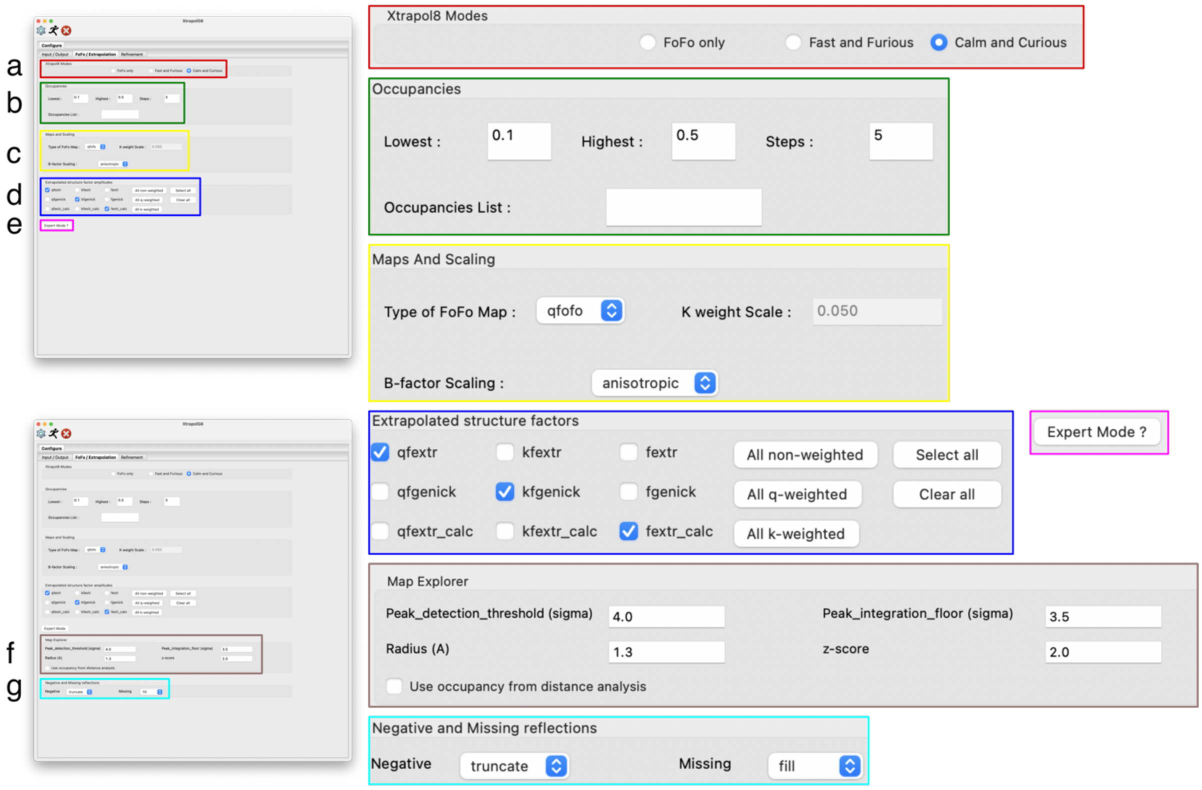

Supplementary Fig. 2 | Panel of the Xtrapol8 GUI to specify parameters and strategies for Fourier difference map and ESFAs calculation. Colored boxes indicate zoom-in at several fields. **a,** users can choose between 3 modes to run Xtrapol8: '*FoFo only*', '*fast and furious*' and *'calm and curious'*. Selection of a specific mode will automatically enable/disable certain parameters to alter. **b,** fields to alter the occupancies to be tested. This can be specified via a minimum and maximum value and number of steps in between or by a list with the values. **c,** in the "Maps and Scaling" fields, the user can precise if the Fourier map should be *q*-weighted, *k*-weighted or non-weighted, as well as how the reference and triggered datasets should be scaled. **d,** field to select the ESFA types. Multiple can be selected in a single run. **e,** more advanced options are offered to more experienced user and concern the parameter for analysis of the difference maps and occupancy estimation **(f)** and the treatment of negative and missing ESFAs **(g)**.

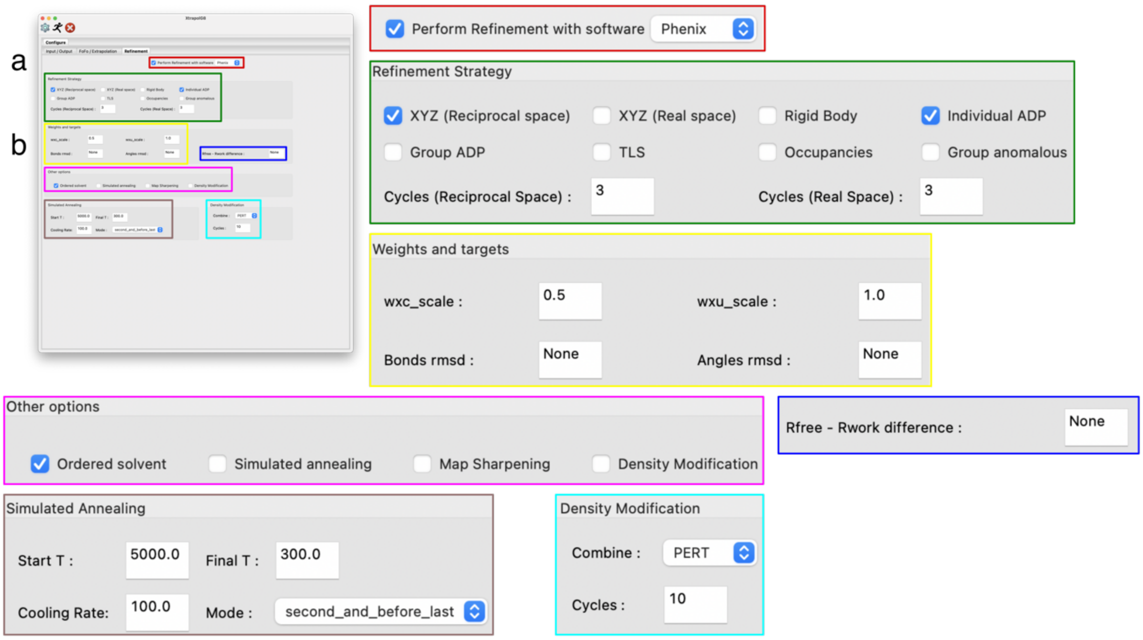

Supplementary Fig. 3 | Panel of the Xtrapol8 GUI concerning refinement with Phenix. Colored boxes indicate zoom-in at several fields. **a,** when the "Perform refinement with" checkbox is ticked, reciprocal and real-space refinement can be carried out using phenix.refine and phenix.real_space_refine. **b,** several options for reciprocal-space refinement with phenix.refine can be altered. The number of refinement cycles in phenix.real_space_refine can also be set.

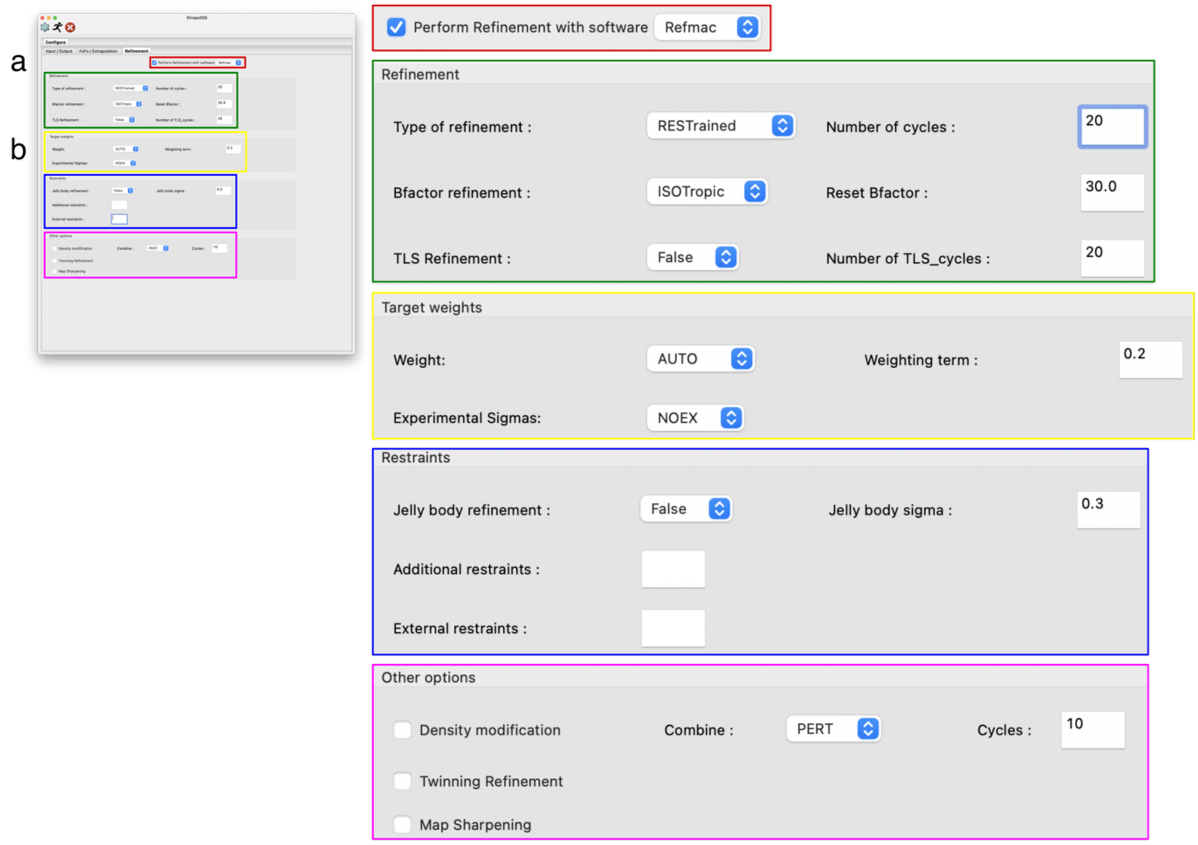

Supplementary Fig. 4 | Alternative panel of the Xtrapol8 GUI concerning refinement with CCP4. Colored boxes indicate zoom-in at several fields. **a,** when the "Perform refinement with" checkbox is ticked, reciprocal and real-space refinement can be carried out using refmac and coot. **b,** several options for reciprocal-space refinement with refmac can be altered.

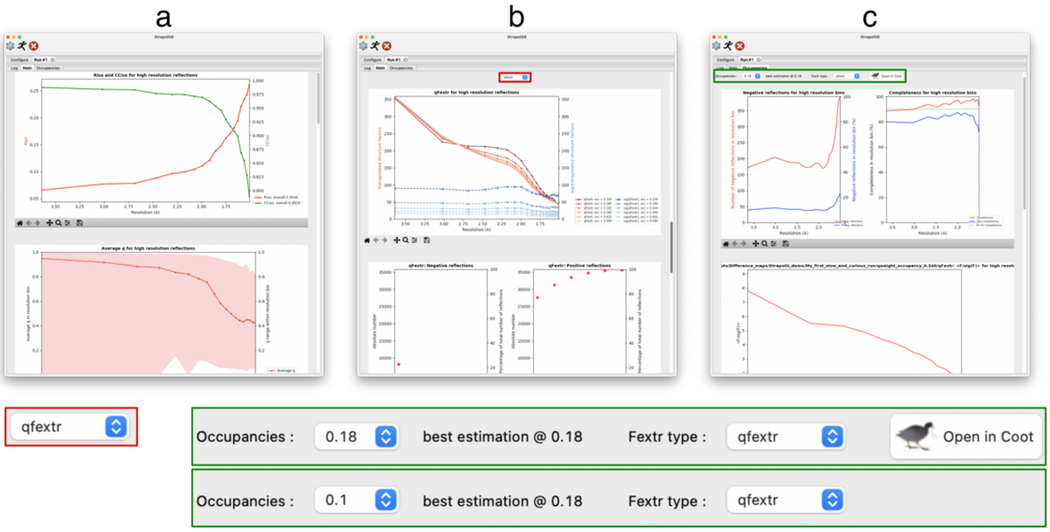

Supplementary Fig. 5 | The results of an Xtrapol8 run using the GUI. Colored boxes indicate zoom-in at several fields. A first tab contains a print of the log-file (not shown). Two other tabs contain the main output or the output considering the occupancies. **a-b,** snippets of the main output tab. **a,** the top part contains general figures, such as the one indicating the isomorphism or the value of the *q*-weights as a function of the resolution. **b,** the bottom part contains information of ESFAs for which the type should be selected via the dropdown menu (red), *e.g.,* signal strength and number of negative and positive ESFAs. **c,** the occupancies tab contains information concerning the ESFAs at each of the tested occupancies, which both have to be selected by a dropdown menu (green). The best estimate of the occupancy for the ESFA type will be indicated and models and maps for that occupancy can be automatically opened in coot.

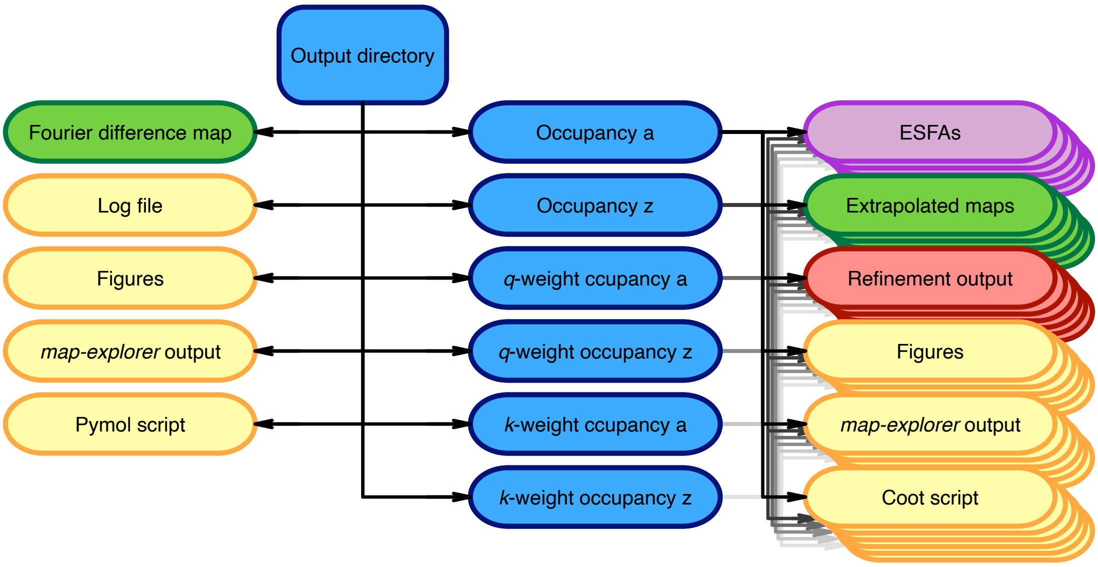

Supplementary Fig. 6 | Schematic representation of the Xtrapol8 output directory. (Sub)directories are shown in blue, electron density maps in green, structure factors in purple, refinement output in red and all other files intended to aid in interpretation and decision making in yellow. Subdirectories are created for each tested occupancy a to z, with the directory name specifying whether Bayesian weighting (*q*/*k*-weighting) or no weighting was used in the calculation of ESFAs.

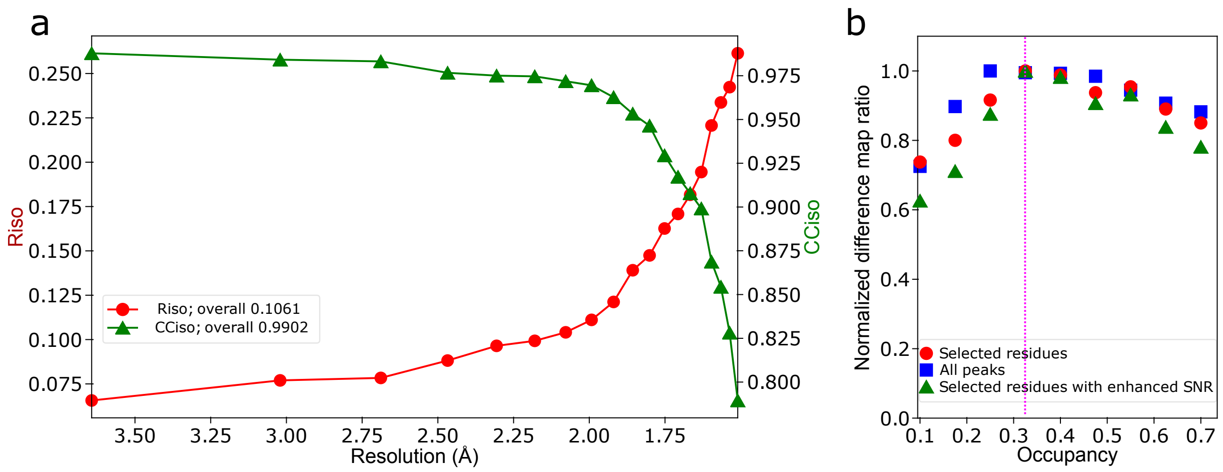

Supplementary Fig. 7 | Xtrapol8 run in *fast-and-furious* mode on the mEos4b data. a, mEos4b isomorphism, expressed as Riso and CCiso, in function of resolution. **b,** mEosb4b occupancy determination after a first Xtrapol8 run in '*fast-and-furious'* mode. The automatically annotated occupancy value is indicated by a magenta dashed line.

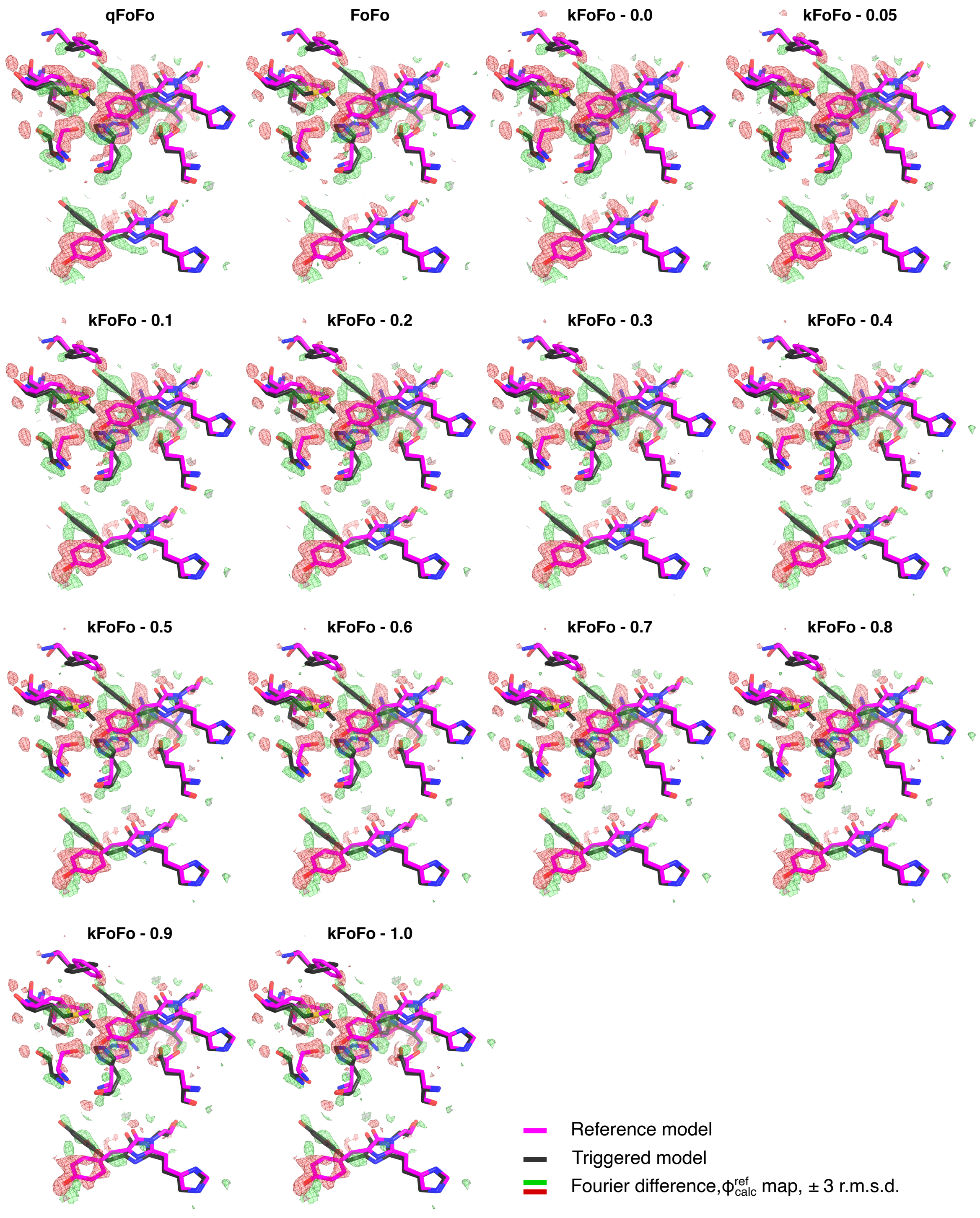

Supplementary Fig. 8 | Effect of different Bayesian weighting schemes to calculate Fourier difference maps for the mEos4b test case. mEos4b chromophore environment (top) and chromophore only (bottom) in the *red-on* and *red-off* state (magenta and gray sticks, respectively) superposed with Fourier difference maps (contoured at ± 3 r.m.s.d.) calculated with the *q*-weighting, no-weighting and *k*-weighting schemes. For *k*-weighting, the additional factor for outlier downweighing is also indicated.

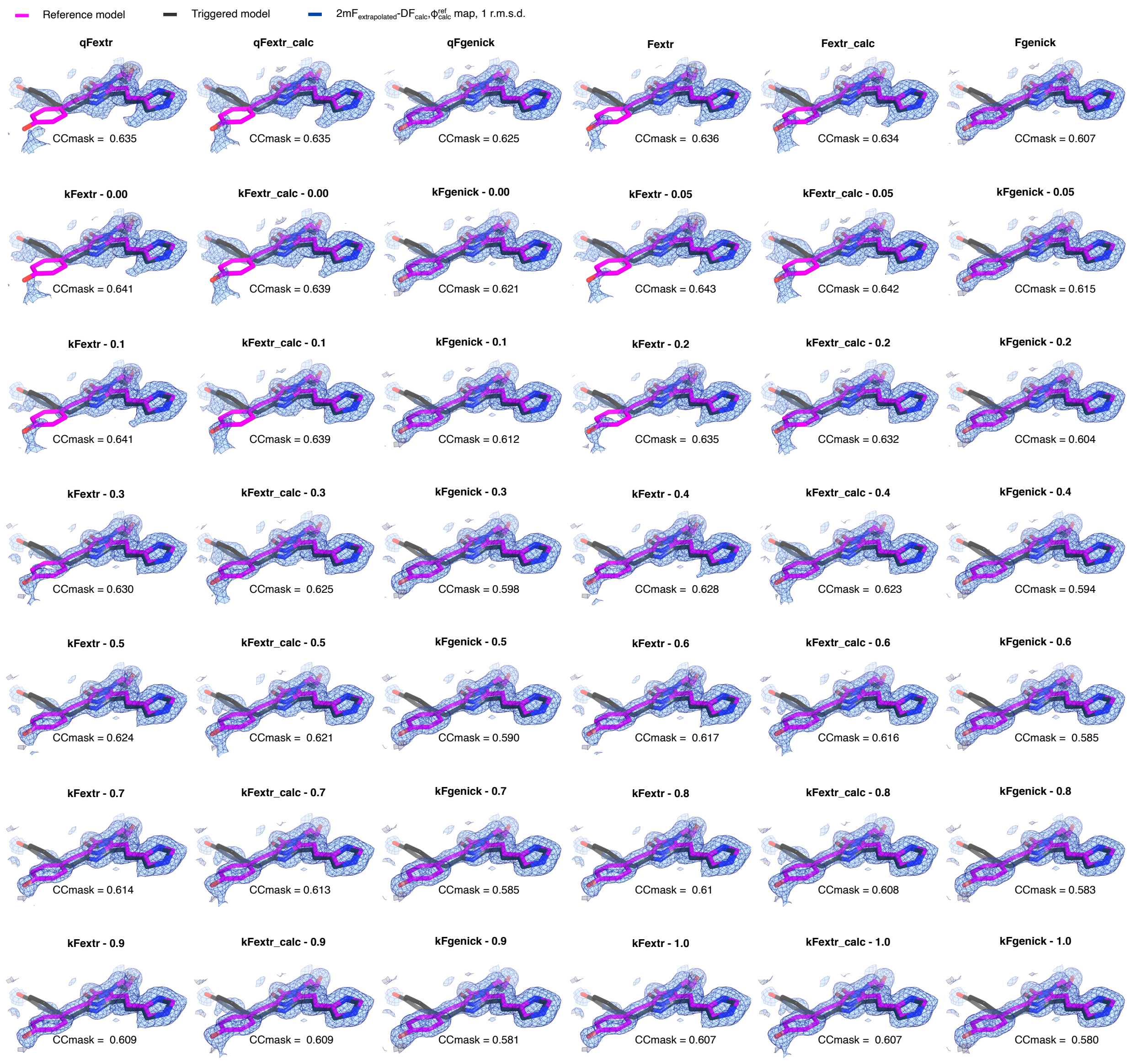

Supplementary Fig. 9 | Illustration of effect of different Bayesian weighting schemes to ESFA calculation for mEos4b. mEos4b chromophore in the *red-on* and *red-off* state (magenta and gray sticks, respectively) superposed on the $2mF_{extrapolated}$-DF_{calc} electron density map calculated for an occupancy of 0.325, contoured at 1 r.m.s.d., for each type of ESFA. In case of *k*-weighting, also the additional scale factor is indicated. For each map, the CCmask is indicated which is calculated using the chromophore and surrounding residues.

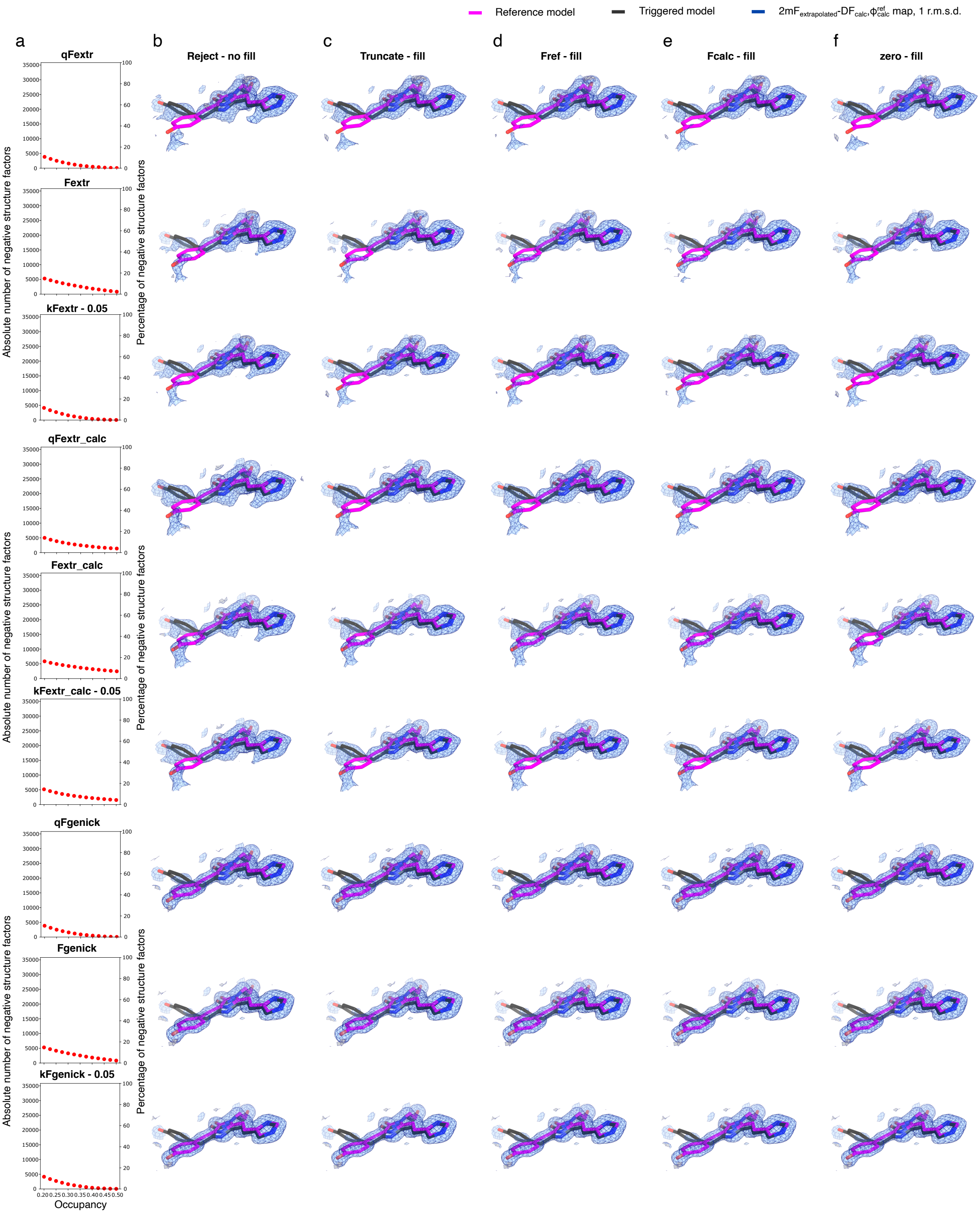

Supplementary Fig. 10 | Effect of different methods to treat negative ESFAs for mEos4b. a, Number of negative ESFAs and 2mF$_{extrapolated}$-DF$_{calc}$ electron density map calculated for an occupancy of 0.325 using different ways to treat negative ESFAs **(b-f)** and ESFA calculation schemes, contoured at 1 r.m.s.d. for the mEos4b chromophore. For *k*-weighting an outlier scale factor of 0.05 was used.

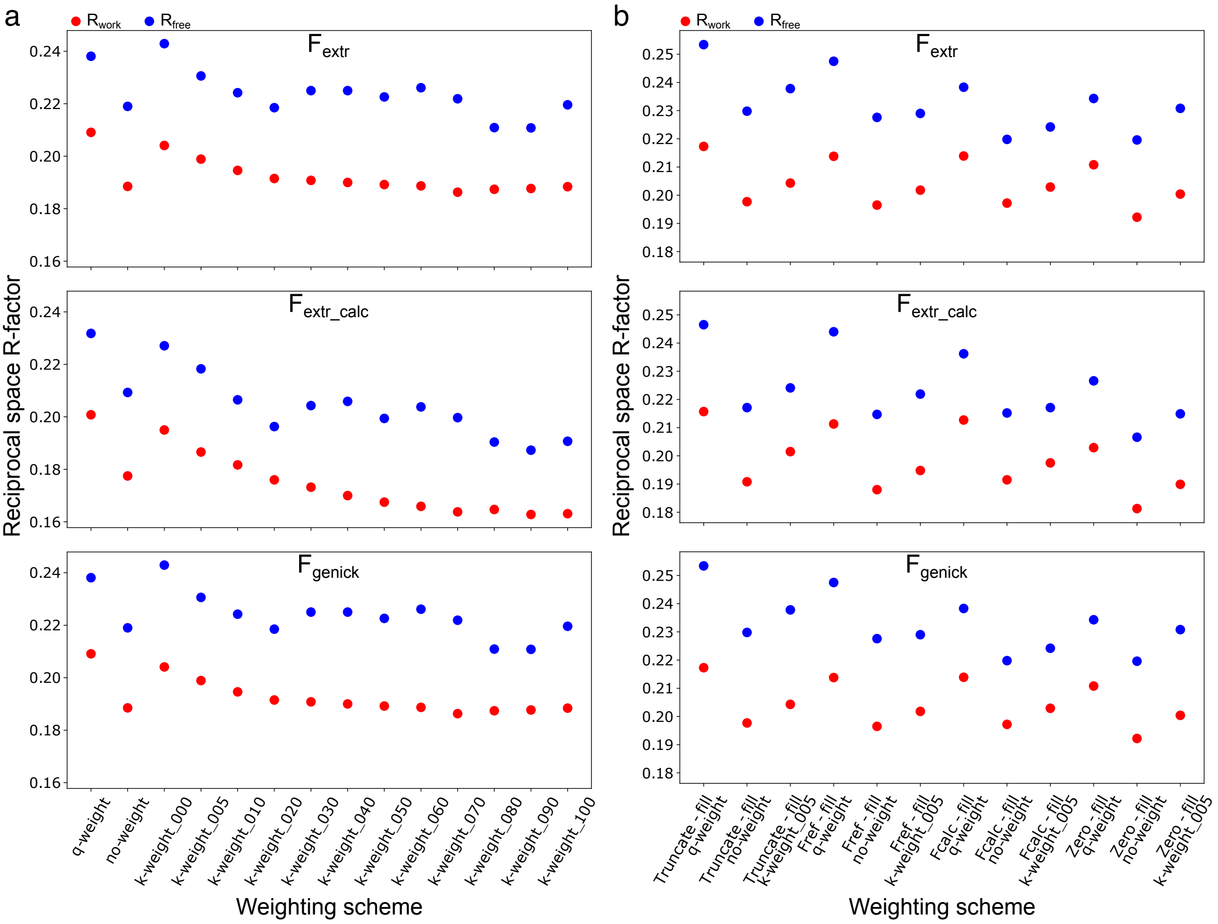

Supplementary Fig. 11 | Refinement R-factors for mEos4b with different Bayesian weighting schemes for the calculation of ESFAs and the various treatments of negative ESFAs. R_{work} (red) and R_{free} (blue) values after reciprocal-space refinement of the published red model (PDB entry 6GP1) in various types of ESFAs and different weighting schemes using the *refiner.py* script. **a,** negative ESFAs were rejected and R-factors are reported for *q*-weighting, no-weighting and *k*-weighting with various *k*-weighting outlier scale factors. **b,** the truncate-, $F_{obs}^{reference}$-, $F_{calc}^{reference}$- and zero-methods are used to estimate negative ESFAs that are *q*-weighted, non-weighted or *k*-weighted (with outlier scale factor of 0.05).

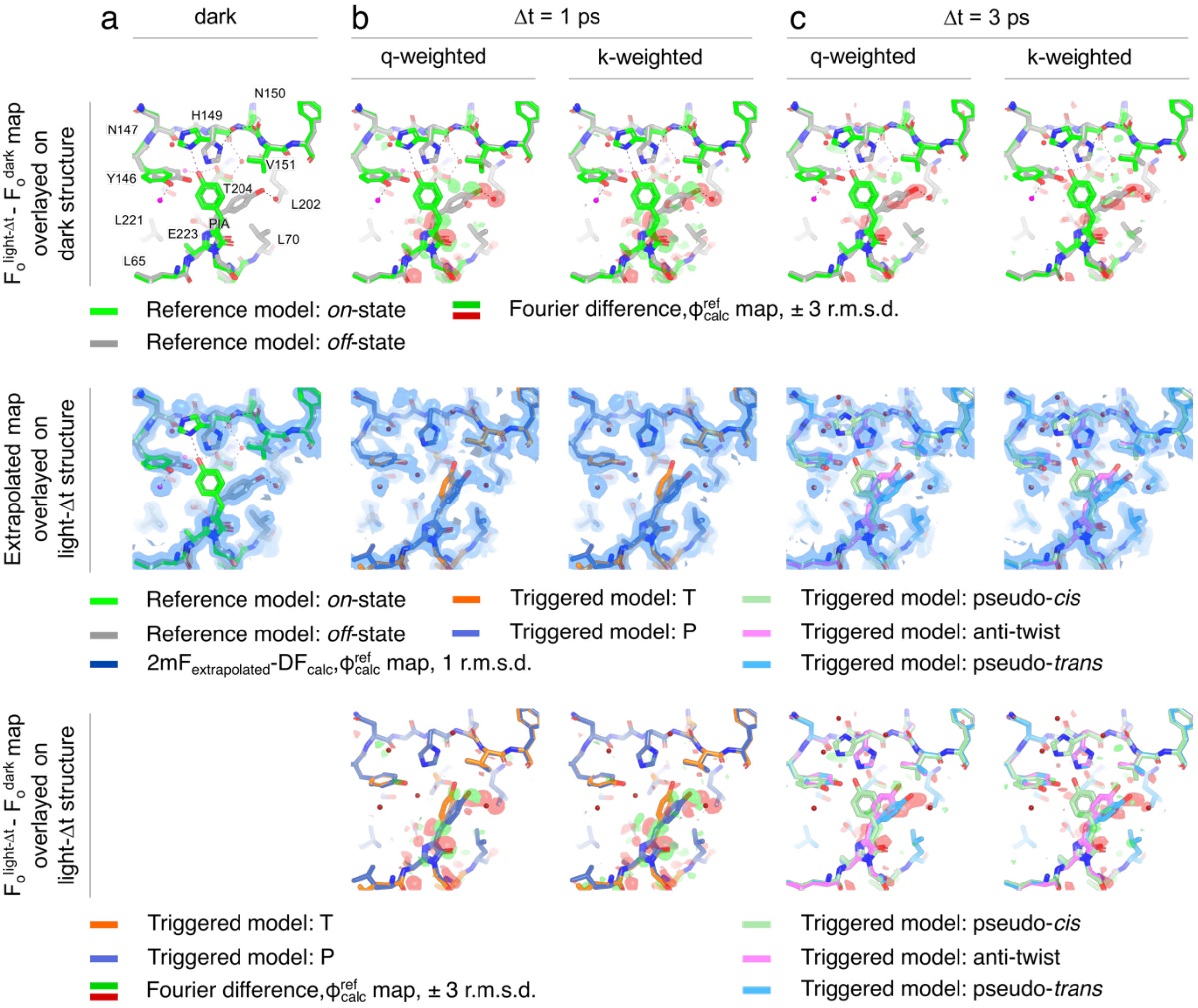

Supplementary Fig. 12 | Summary of Xtrapol8 outcome on the extraction of ps lived intermediates states in rsEGFP2. a, A dataset collected on crystalline rsEGFP2 in the OFF state was used as the reference dataset (laser-OFF), while two datasets collected at 1 **(b)** and 3 ps **(c)** post-pumping by a UV laser fs-pulse served as the triggered datasets (laser-ON). The top row shows the laser-OFF state structure, which features 90% of molecules in the fluorescent OFF-state (grey) and the remainder in the fluorescent ON-state (green), with the *q*/*k*-weighted Fourier difference maps overlaid. The middle row shows the $2mF_{obs}$-DF_{calc} (laser-OFF) and *q*/*k*-weighted $2mF_{extrapolated}$-DF_{calc} (laser-ON) electron density maps contoured at 1 r.m.s.d. overlaid on the refined laser-ON models (conformers are depicted with different colors). The bottom row shows a superposition of the laser-ON models superposed on the *q*/*k*-weighted Fourier difference maps contoured at ± 3 r.m.s.d.

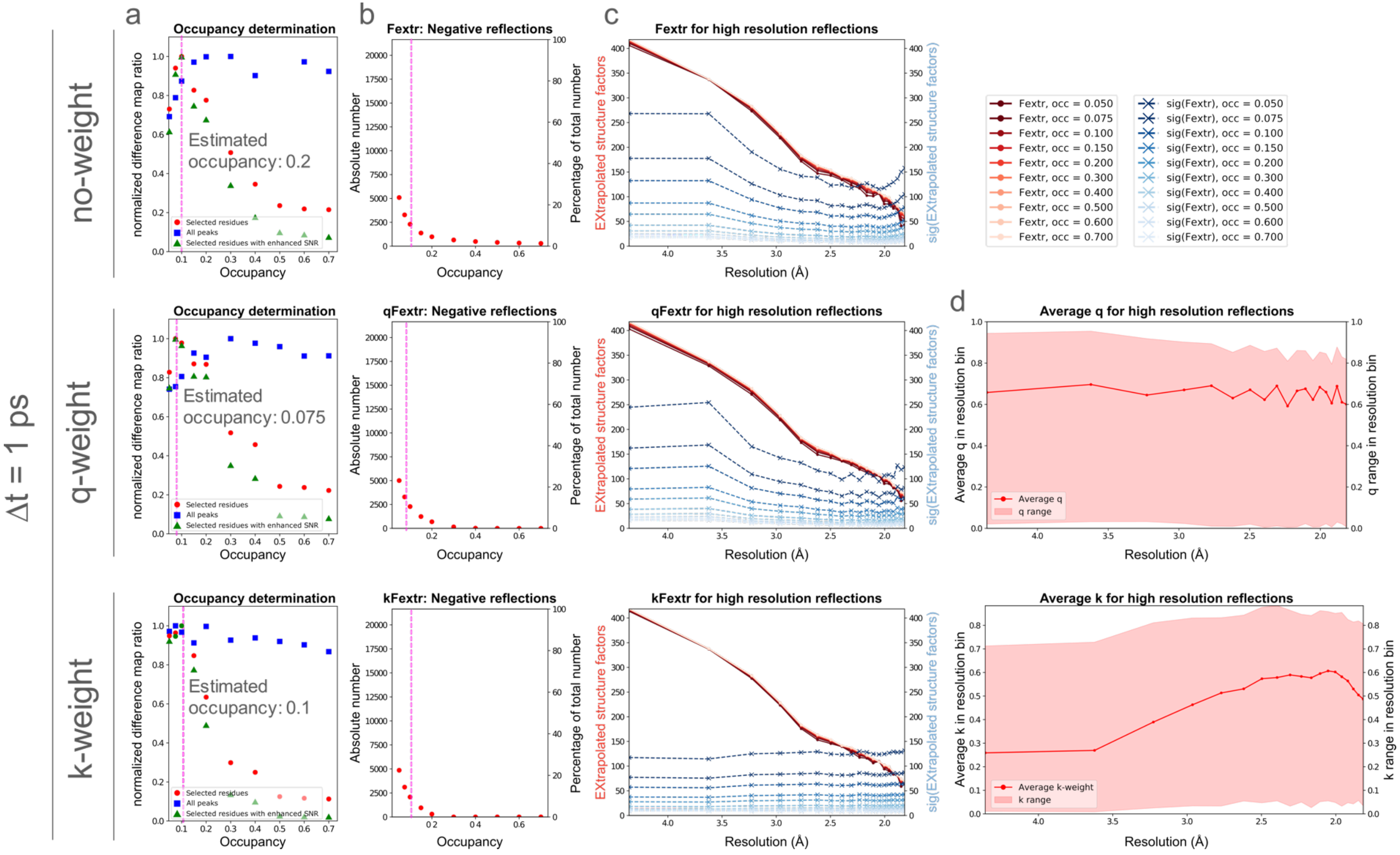

Supplementary Fig. 13 | Effect of Bayesian-statistics weighting of ESFAs computed for various occupancies from the reference and Δt=1 ps data. The top, middle and bottom rows show results with no weighting, *q*-weighting or *k*-weighting of EFSAs. **a**, Bayesian weighting of ESFAs hardly influences the outcome of occupancy determinations carried out using the *difference-map* method. **b**, Bayesian weighting of ESFAs only slightly reduces the amount of negative ESFAs. In (**a**) and (**b**), the occupancies determined by the difference map method are highlighted by a pink shaded line. **c**, Plots of mean ESFA (red to orange) and corresponding σ(ESFA) (blue) as a function of resolution. **d**, Plots of *q*-weights and *k*-weights average value and range of values as a function of resolution. The shaded area shows the range of *q*-weights and *k*-weights.

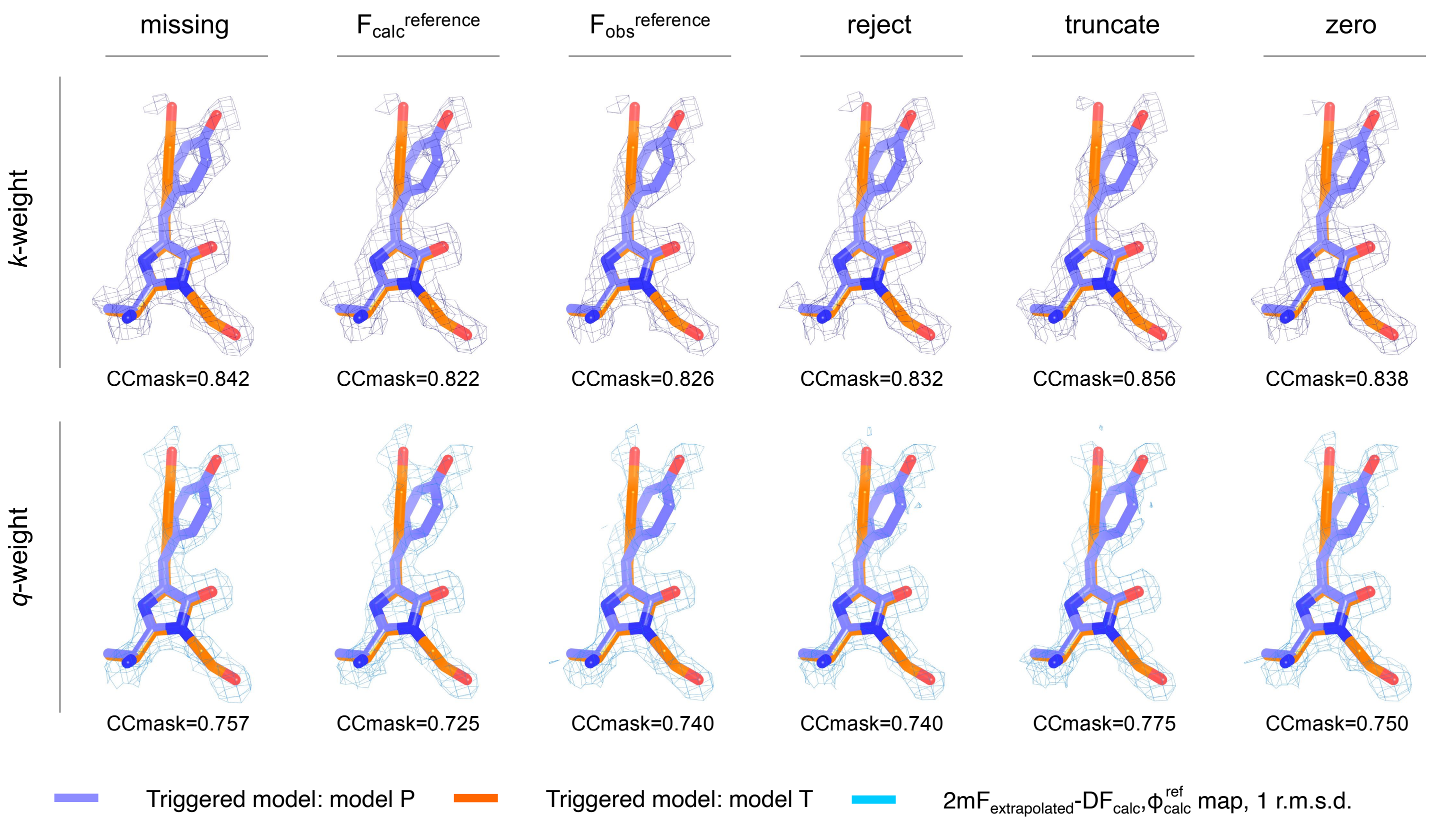

Supplementary Fig. 14 | Illustration of the effect of negative-ESFA handling on the extrapolated maps calculated for the Δt = 1ps time delay. The initial $2mF_{extrapolated}$-DF_{calc} maps (contour level 1 r.m.s.d.) obtained using the various negative-ESFAs handling-approaches implemented in Xtrapol8 and a triggered-state occupancy of 7.5 % are overlaid on the laser-ON model refined on the basis of the *k*-weighted ESFA produced with the truncate method. For clarity, only the chromophore and the corresponding extrapolated electron density are shown. The overall CCmask of the model is indicated, which was calculated using all atoms in the models. The CCmask for the *q*-weighted extrapolated maps and models is lower than that for the *k*-weighted counterparts, possibly due to damping of structural differences due by the *k*-weighting procedure, as compared to *q*-weighting. Regardless, the CCmask is higher for the extrapolated maps calculated from ESFAs produced with the truncate method, showcasing the importance of rescuing negative ESFAs. The CCmask value between the reference model and laser-OFF map equals 0.951.

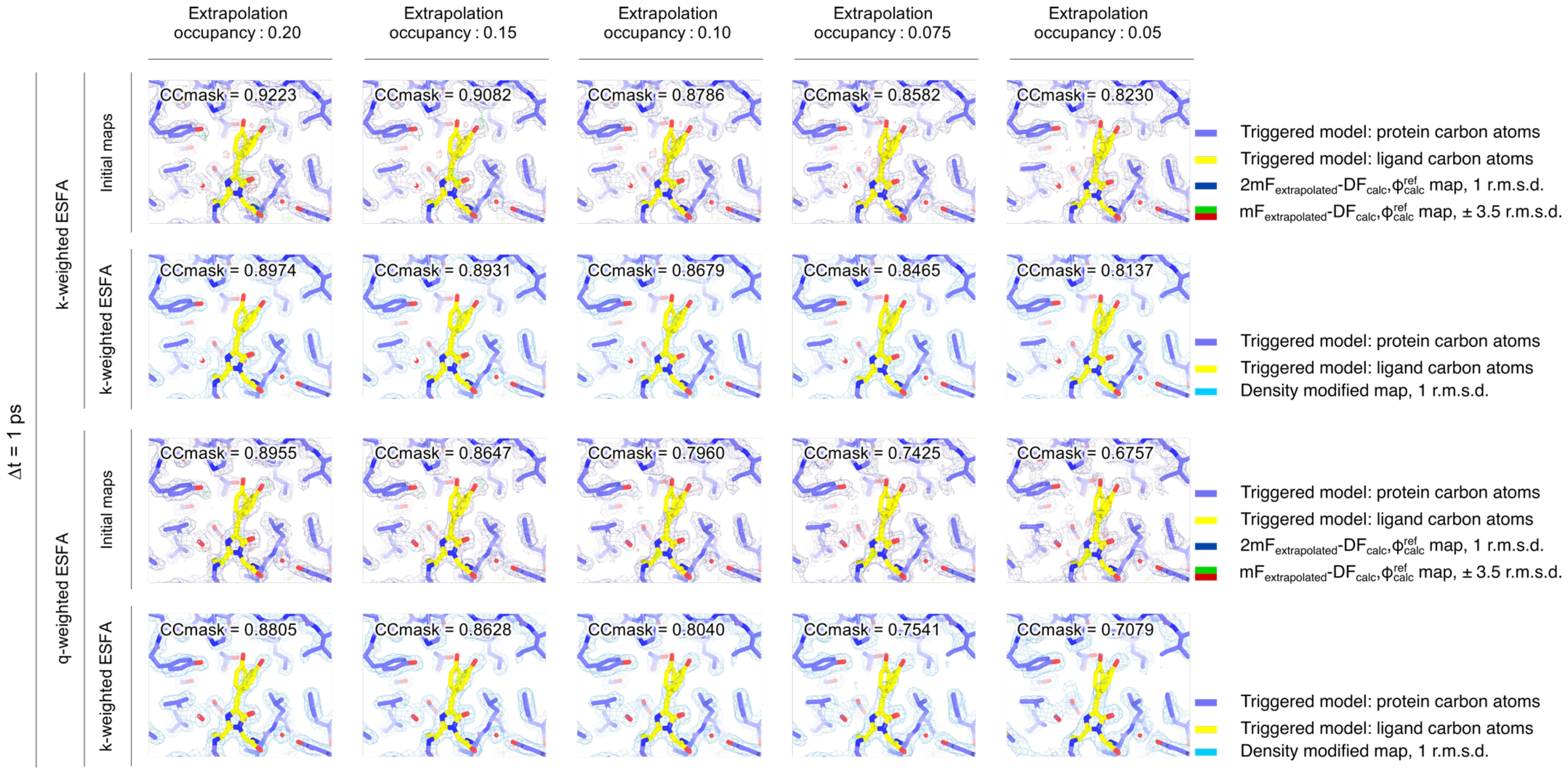

Supplementary Fig. 15 | Evolution of features and noise in the *k*-weighted and *q*-weighted $2mF_{extrapolated}$-DF_{calc} initial and density modified maps computed at various extrapolation occupancies from the laser-OFF and laser-ON Δt=1 ps datasets. While density modification slightly benefits map quality in the case of *q*-weighted ESFAs, it slightly decreases it in the case of *k*-weighted ESFAs. Thus, while it sounds reasonable that density modification should improve the quality of the maps, it is not always the case in practice and hence the opportunity of basing real-space refinement on these should be verified on a case-by-case basis.

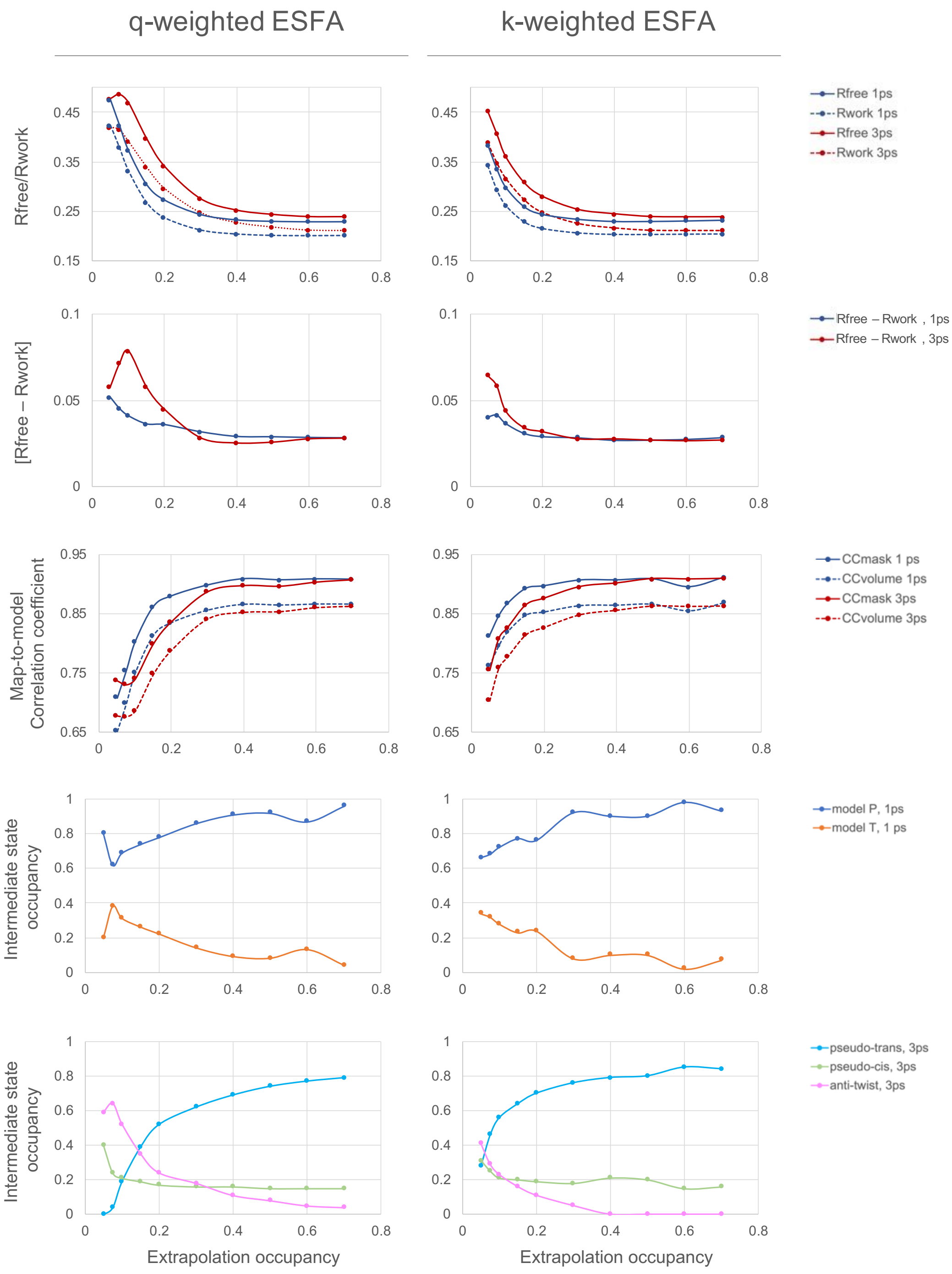

Supplementary Fig. 16 | Automatic refinement of the laser-ON models of rsEGFP2 using the *refiner.py* script of Xtrapol8. For both the Δt=1 ps and Δt=3 ps laser-ON models, automatic refinement was unable to account for the large change in chromophore conformation, hence, manual intervention was required. For each time delay, a conservative model was produced on the basis of features in the *k*-weighted $2mF_{extrapolated}$-DF_{calc} map computed for an occupancy of 0.2, which was then subjected to refinement against *q*-weighted (left column) and *k*-weighted ESFAs (right column) calculated for all occupancies. **a,** R_{work} and R_{free} values of the extrapolated structures after 10 cycles of reciprocal-space refinement including positional and occupancy refinements; results are shown for the Δt=1 ps (blue) and the Δt=3 ps (red) datasets. **b,** R_{work} - R_{free} difference values; to ensure that this value remains in the 3-5% range even at lower occupancies, the wxc_scale value was set to 0.0000002. **c,** CCmask and CCvolume values after real-space refinement in the reciprocal-space refined extrapolated density maps. **d-e,** Refined occupancies determined for the two conformers upon refinement against *q*-weighted and *k*-weighted ESFAs for the Δt=1 ps **(d)** and Δt=3 ps **(e)** datasets.

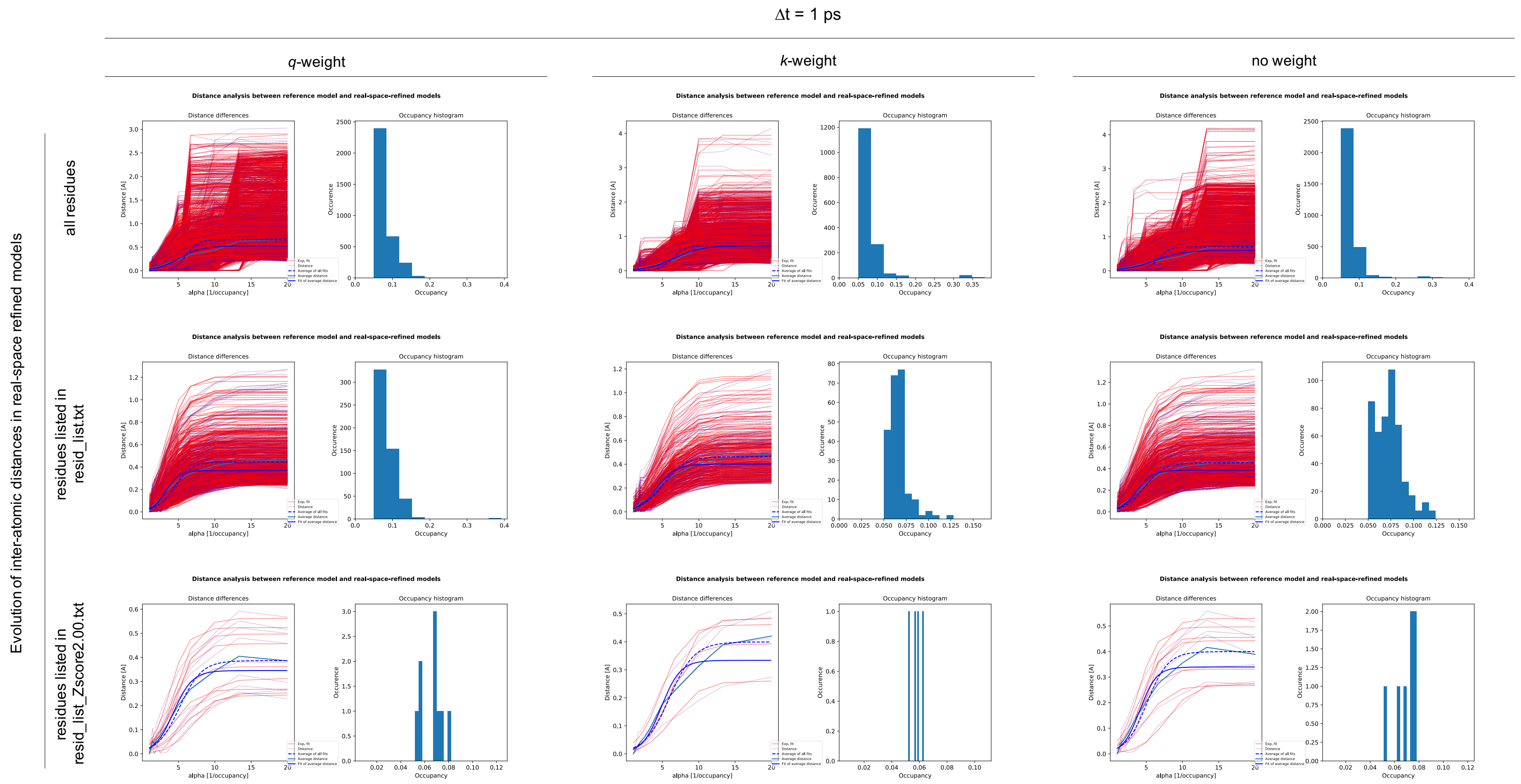

Supplementary Fig. 17 | Occupancy determination for the Δt = 1 ps dataset based on the distance analysis method. The distance analysis method estimates the correct occupancy by fitting the evolution of interatomic distances as a function of all tested occupancies with a sigmoid, and then retrieving the occupancy value for which 99% of the plateau is reached. The left graph on each panel shows the individual interatomic distances (purple lines), sigmoidal fits (red lines), the average of all fits (blue dashed line), the average of all distances (teal line) and the fit of the average (full blue line). The right graph shows the distribution of occupancy values obtained from the individual fits.

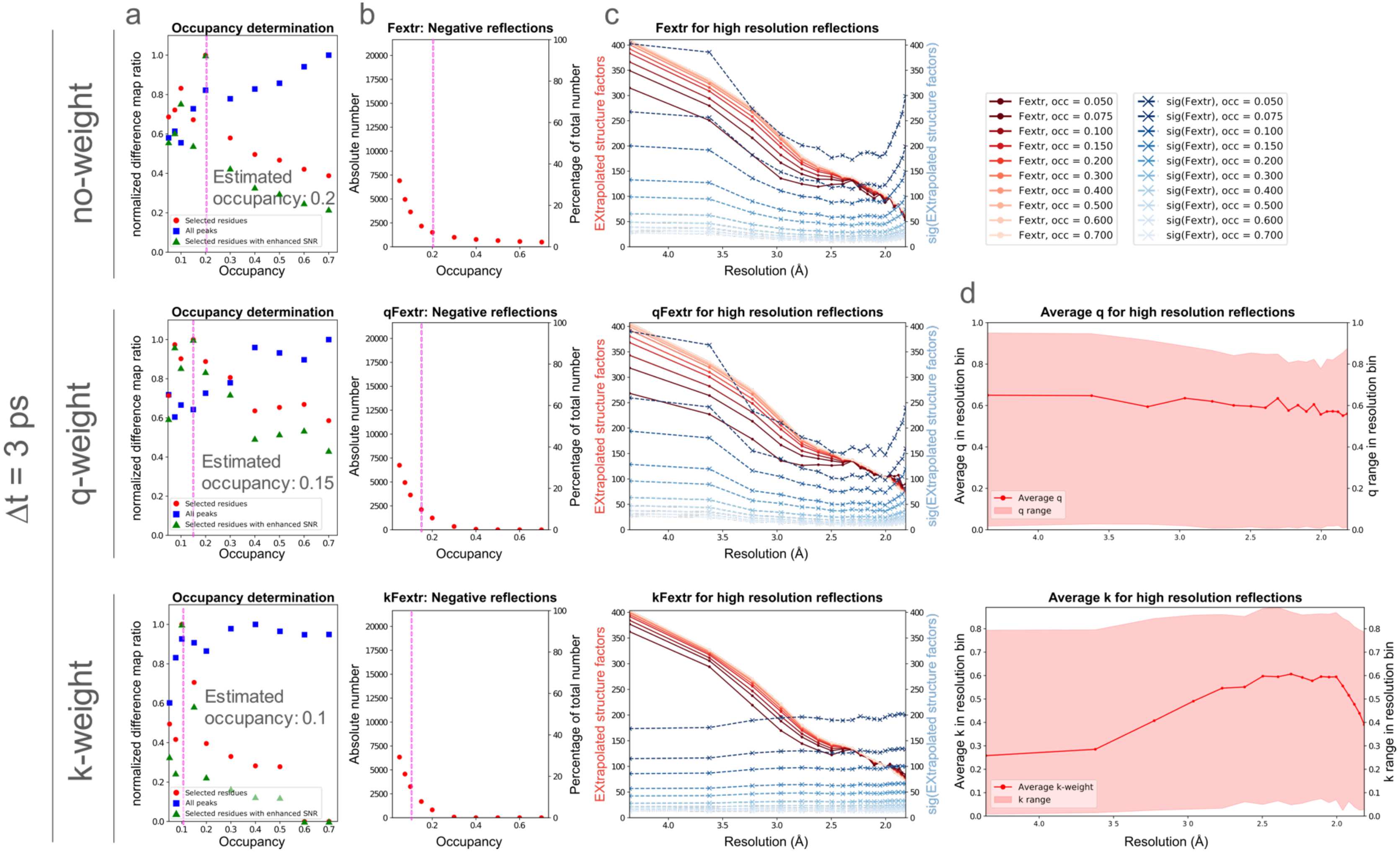

Supplementary Fig. 18 | Effect of Bayesian-statistics weighting of ESFAs computed for various occupancies from the reference and Δt=3 ps data. The top, middle and bottom rows show results with no weighting, *q*-weighting or *k*-weighting of EFSAs. **a,** Bayesian weighting of ESFAs hardly influences the outcome of occupancy determinations carried out using the difference map method. **b,** Bayesian weighting of ESFAs only slightly reduces the amount of negative ESFAs. In **(a)** and **(b)**, the occupancies determined by the difference map method are highlighted by a pink shaded line. **c,** Plots of mean ESFA (red to orange) and corresponding σ(ESFA) (blue) as a function of resolution. Last column: Plots of *q*-weights and *k*-weights average value and range of values as a function of resolution. The shaded area shows the range of *q*-weights and *k*-weights.

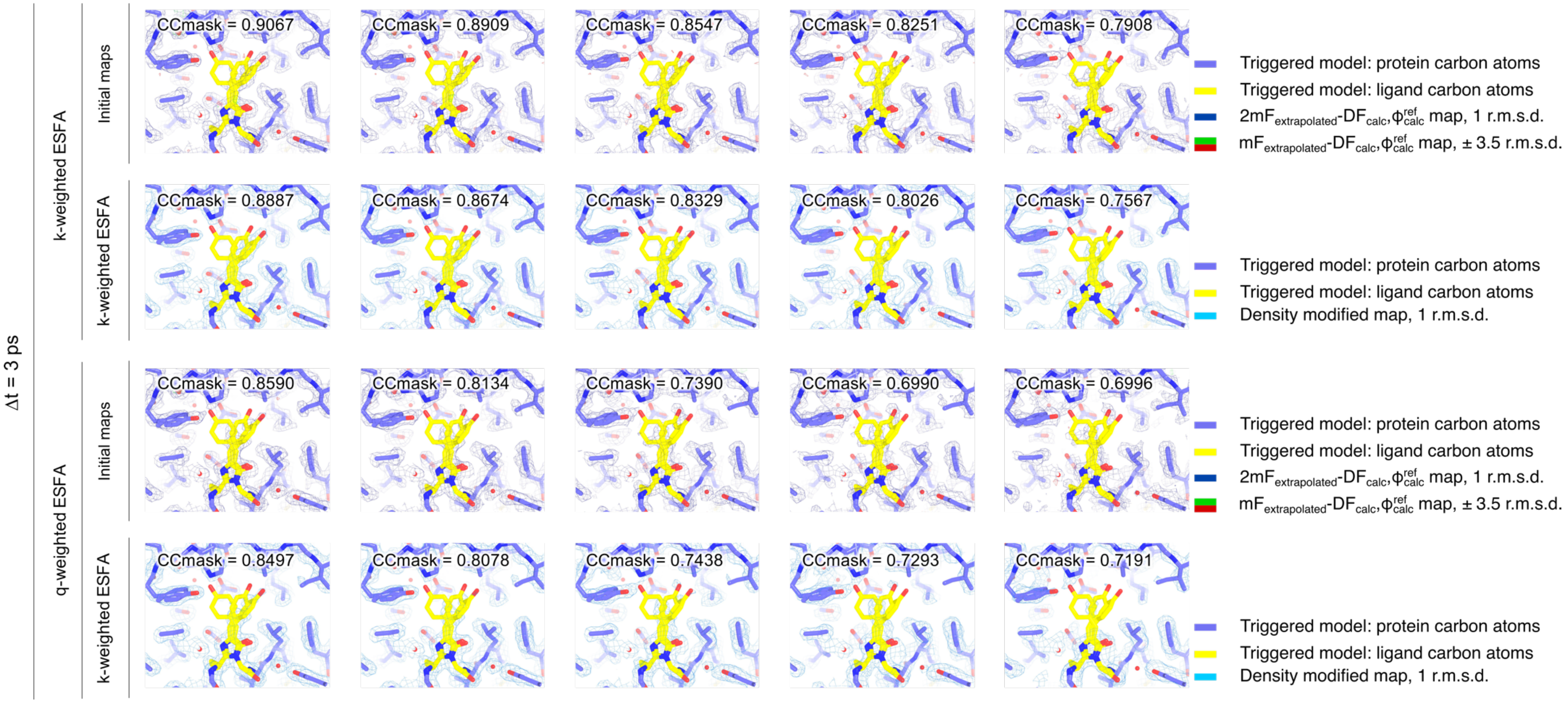

Supplementary Fig. 19 | Evolution of features and noise in the *k*-weighted and *q*-weighted 2mF$_{extrapolated}$-DF$_{calc}$ initial and density modified maps computed at various extrapolation occupancies from the laser-OFF and laser-ON Δt=3 ps datasets. While density modification slightly benefits map quality in the case of *q*-weighted ESFAs, it slightly decreases it in the case of *k*-weighted ESFAs. Thus, while it sounds reasonable that density modification should improve the quality of the maps, it is not always the case in practice and hence the opportunity of basing real-space refinement on these should be verified on a case-by-case basis.

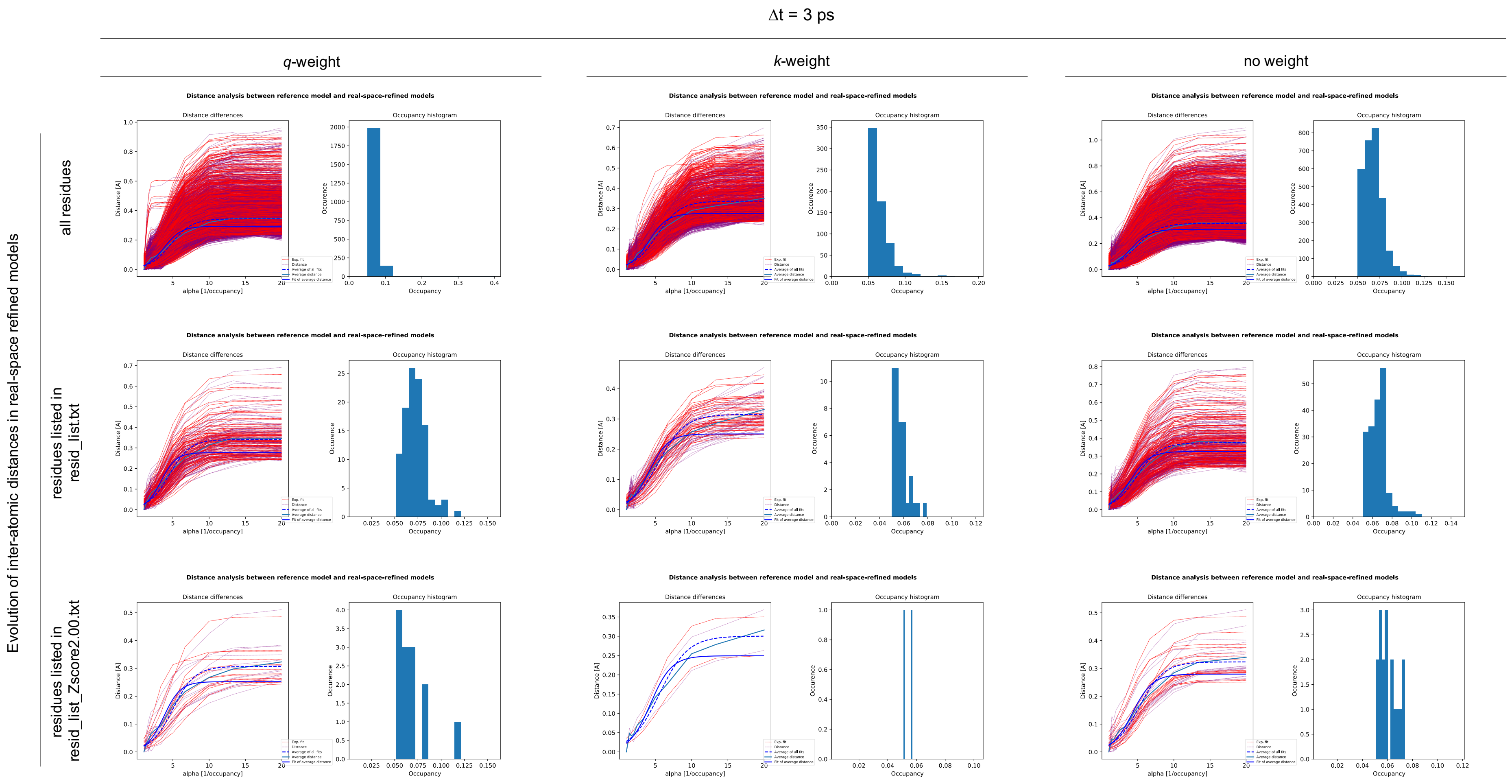

Supplementary Fig. 20 | Occupancy determination for the Δt = 3 ps dataset based on the *distance-analysis* method. The distance analysis method estimates the correct occupancy by fitting the evolution of interatomic distances as a function of all tested occupancies with a sigmoid, and then retrieving the occupancy value for which 99% of the plateau is reached. The left graph on each panel shows the individual interatomic distances (purple lines), sigmoidal fits (red lines), the average of all fits (blue dashed line), the average of all distances (teal line) and the fit of the average (full blue line). The right graph shows the distribution of occupancy values obtained from the individual fits.

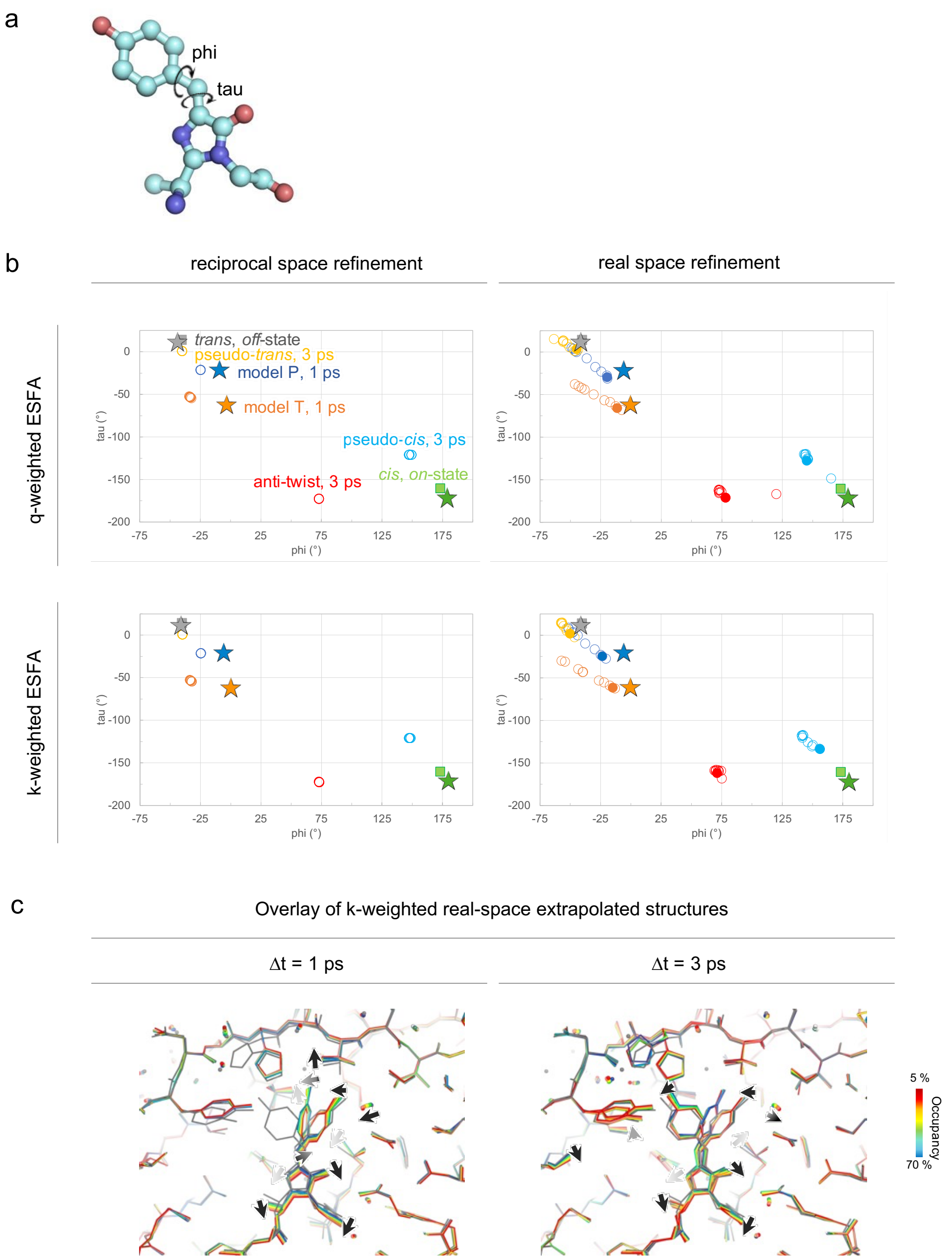

Supplementary Fig. 21 | Chromophore conformations observed in the static and time-resolved structures of rsEGFP2. **a**, The conformation of the chromophore of a fluorescent protein, including rsEGFP2, is defined by two dihedral angles, tau and phi. **b**, Changes in chromophore conformation, and the influence on those of Bayesian weighting and refinement, are best visualized in tau vs. phi plots. Squares point to the conformations refined for the static *on*-state (*cis*-anionic) and *off*-state (*trans*-protonated) structures using conventional reciprocal-space refinement. Circles indicate the conformations automatically refined in the reciprocal-space and the real-space, respectively, from ESFAs calculated at various occupancies for the Δt=1 and 3 ps structures, using the *refiner.py* script of Xtrapol8. The filled circles indicate results for an occupancy of 0.1. For comparison, the conformations reported in Ref[13] are indicated by stars. The figure highlights that use of strong restraints in reciprocal-space refinement, sometimes needed to avoid the R_{work}-R_{free} gap to increase, may limit the movement of atoms. Changes in electron density will however be visible in the resulting maps, so that subsequent real-space refinement will allow to finely model the conformations adopted by the chromophore. **c,** Structures refined at various extrapolation occupancies. The models are coloured from cold to hot, with blue and red pointing to the structures refined at the highest and lowest extrapolation occupancies, respectively. It is notable that past a given occupancy, here ~0.1, models hardly vary.

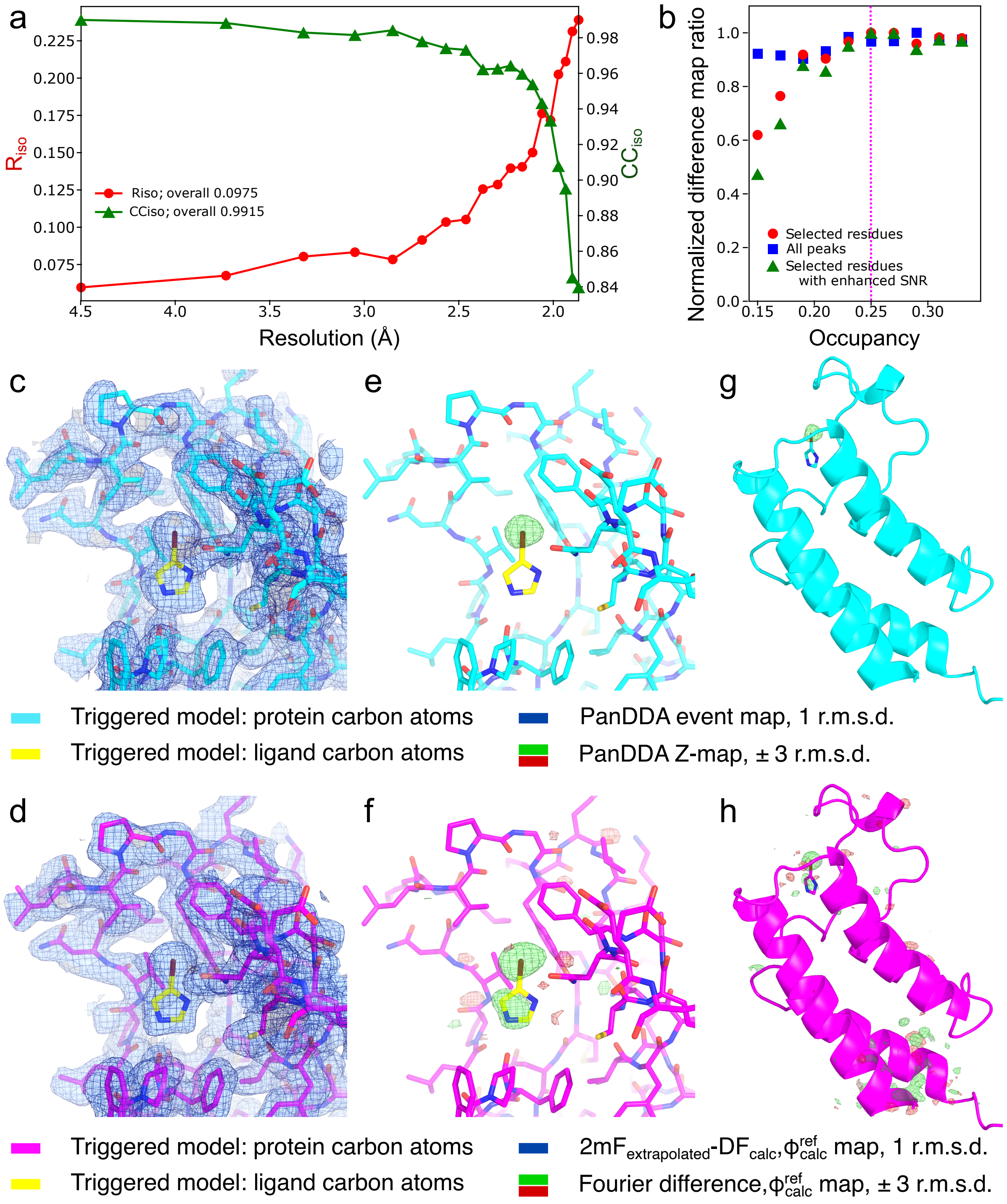

Supplementary Fig. 22 | Comparison of Xtrapol8 with PanDDA on BAZ2BA-x538. a, Isomorphism (Riso and CCiso) between the BAZ2BA-x538 and BAZ2BA-x645 data sets up to a high resolution of 1.8 Å. **b,** occupancy with the *difference-map* method for the ligand using structure factor extrapolation (type: *q*Fextr). **c,** PanDDA event map for the ligand and surroundings, contoured at 1 r.m.s.d. **d,** *q*-weighted $2mF_{extrapolated}$-DF_{calc} electron density map with an occupancy of 0.25 for the ligand and environment, contoured at 1 r.m.s.d. **e,** PanDDA z-map for the ligand, contoured at ± 4 r.m.s.d. **f,** *q*-weighted Fourier difference map for the ligand, contoured at ± 4 r.m.s.d. **g,** PanDDA z-map for the complete protein, contoured at ± 4 r.m.s.d. **h,** *q*-weighted Fourier difference map for the complete protein, contoured at ± 4 r.m.s.d. The model in cyan and magenta are the BAZ2BA-x645 model wherein the 1,2-ethanediol is replaced by the 4-bromoimidazole ligand (yellow) based on the PanDDA event or on extrapolated electron density maps, respectively.

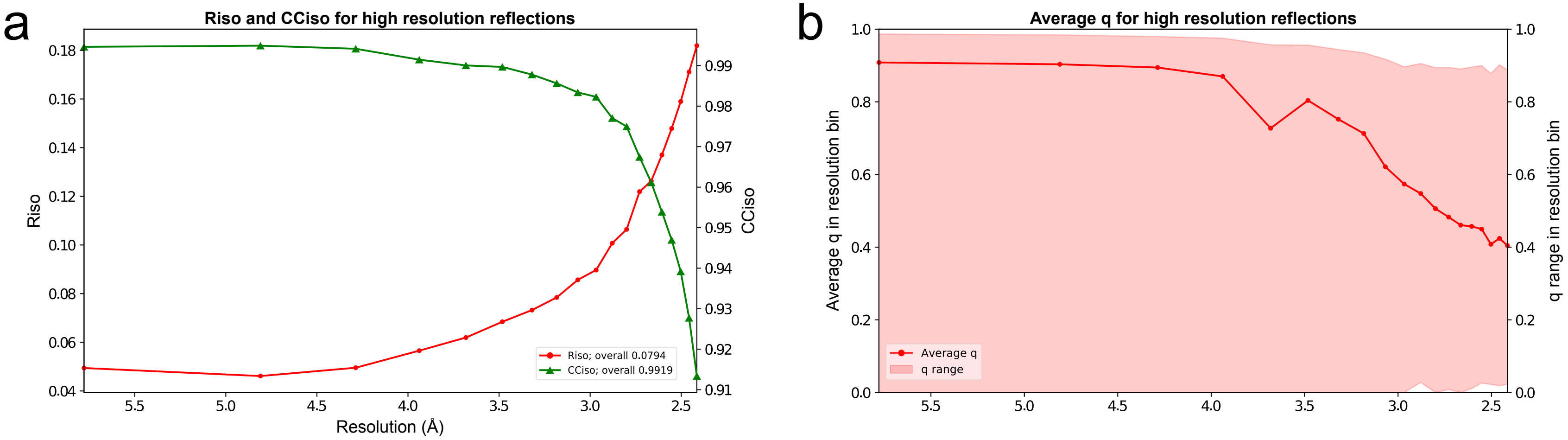

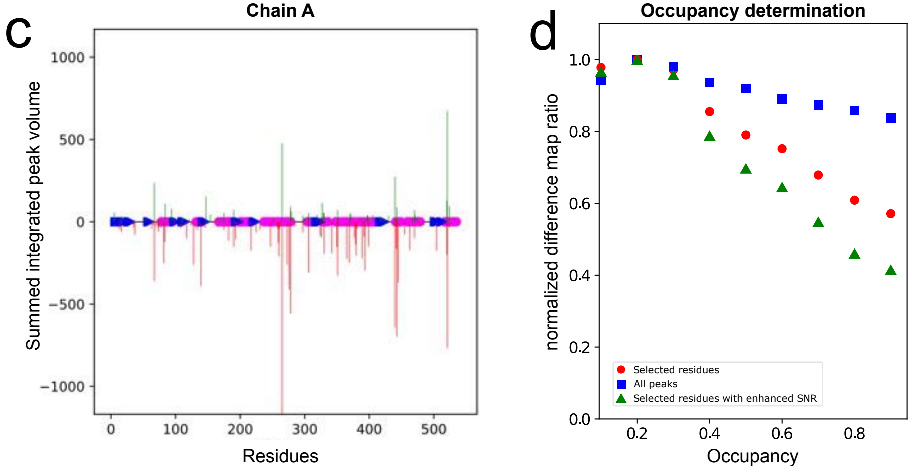

e

Type of acidic residue		Density visible at 0.8 σ in $2qmF_{ext}^{2vjb-2vja} - DFc$ maps	
		Monomer A	Monomer B
Aspartate	Number of residues	6	9
	Residues number	72, 93, 172, 182, 326, 397	72, 93, 172, 182, 326, 377, 380, 381, 397
	Residues consistently preserved from damage	72, 93, 172, 182, 326, 397	
Glutamate	Number of residues	7	8
	Residues number	5, 82, 92, 443, 462, 499	5, 73, 92, 139, 163, 240, 306, 443, 455, 462
	Residues consistently preserved from damage	5, 92, 443, 462	

Supplementary Fig. 23 | Data quality assessment and analysis of structural differences in the Shoot-and-Trap test case using the data sets collected at 100K. a, R_{iso} and CC_{iso} values indicating the isomorphism between the 2VJA and 2VJB data sets. **b,** *q*-weight in function of the resolution. The shaded area shows the *q*-weights range. **c,** Distribution of Fourier difference peaks throughout chain A of the asymmetric unit. α-helices and β-sheets are depicted by magenta rods and blue triangles, respectively. **d,** Occupancy determination using the *difference-map* method; occupancy of the triggered state is predicted to be 0.2. **e,** Analysis of X-ray induced decarboxylation of acidic residues throughout the two chains (A, B) in the asymmetric unit. Numbers highlighted in green refer to residues which are decarboxylated or preserved from decarboxylation in both monomers of the asymmetric unit dimer.

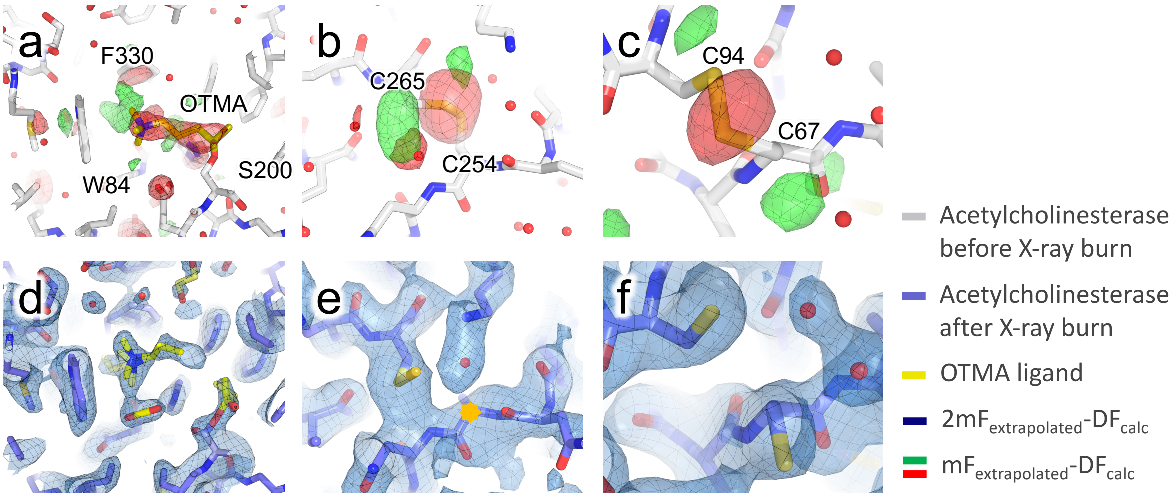

Supplementary Fig. 24 | Fourier difference and extrapolated electron density maps for the Shoot-and-trap experiment performed on the crystalline complex of acetylcholinesterase with a non-hydrolysable substrate analogue at 100 K. The Fourier difference maps in **a-c** are contoured at ± 3 r.m.s.d., the 2mF$_{extrapolated}$-DF$_{calc}$ electron density maps in **d-f** are contoured at 1 r.m.s.d. The reference model collected at 3 MGy (2VJA) is depicted in gray, the model at 9 MGy (2VJB) in blue, except for the OTMA ligand and its radiolitic product which are shown in yellow. **a-c,** Specific radiation damage, manifested by negative peaks in the q-weighted $F_{obs}^{2VJB} - F_{obs}^{2VJA}$ Fourier difference map, is observed on the substrate analogue, carboxylate groups and disulfide bridges, respectively, indicating cleavage of the covalent bond to the protein, decarboxylation and rupture of disulfide bridges. Positive peaks are also seen that point to a reorientation of radiolysis-products in the active site (CO_2, carbocholine, ethanal) and throughout the protein (CO_2, cysteines). **d,** the q-weighted 2mF$_{extrapolated}$-DF$_{calc}$ extrapolated map clarifies the outcome of the radiolysis at 100 K of the covalently bounded substrate analogue, revealing how CO_2, carbocholine and ethanal reorient in the active site. **e-f,** Extrapolated maps also clearly evidence X-ray-induced rupture of disulfide bridges.

Supplementary References

1. White, T. A. Post-refinement method for snapshot serial crystallography. *Philos Trans R Soc L. B Biol Sci* **369**, 20130330 (2014).

2. Kabsch, W. XDS. *Acta. Crystallogr. D* **66**, 125–132 (2010).

3. Evans, P. R. & Murshudov, G. N. How good are my data and what is the resolution? *Acta Crystallogr D Biol Crystallogr* **69**, 1204–1214 (2013).

4. Afonine, P. V *et al.* Towards automated crystallographic structure refinement with phenix. refine. *Acta Crystallogr. D* **68**, 352–367 (2012).

5. Afonine, P. V *et al.* Real-space refinement in PHENIX for cryo-EM and crystallography. *Acta Crystallogr D Struct Biol* **74**, 531–544 (2018).

6. Liebschner, D. *et al.* Macromolecular structure determination using X-rays, neutrons and electrons: recent developments in Phenix. *Acta Crystallogr D Struct Biol* **75**, 861–877 (2019).

7. Ren, Z. *et al.* A molecular movie at 1.8 A resolution displays the photocycle of photoactive yellow protein, a eubacterial blue-light receptor, from nanoseconds to seconds. *Biochemistry* **40**, 13788–13801 (2001).

8. Greisman, J. B., Dalton, K. M. & Hekstra, D. R. reciprocalspaceship : a Python library for crystallographic data analysis . *J. Appl. Crystallogr.* **54**, (2021).

9. Genick, U. K. *et al.* Structure of a protein photocycle intermediate by millisecond time-resolved crystallography. *Science (80-.).* **275**, 1471–1475 (1997).

10. Genick, U. K. Structure-factor extrapolation using the scalar approximation: theory, applications and limitations. *Acta Crystallogr D Biol Crystallogr* **63**, 1029–1041 (2007).

11. Grosse-Kunstleve, R. W., Sauter, N. K., Moriarty, N. W. & Adams, P. D. The Computational Crystallography Toolbox: crystallographic algorithms in a reusable software framework. *J. Appl. Crystallogr.* **35**, 126–136 (2002).

12. El Khatib, M., Martins, A., Bourgeois, D., Colletier, J. P. & Adam, V. Rational design of ultrastable and reversibly photoswitchable fluorescent proteins for super-resolution imaging of the bacterial periplasm. *Sci. Rep.* **6**, 18459 (2016).

13. Coquelle, N. *et al.* Chromophore twisting in the excited state of a photoswitchable fluorescent protein captured by time-resolved serial femtosecond crystallography. *Nat. Chem.* **10**, 31–37 (2018).

14. Pearce, N. M. *et al.* A multi-crystal method for extracting obscured crystallographic states from conventionally uninterpretable electron density. *Nat Commun* **8**, 15123 (2017).

15. Pearce, N. M., Bradley, A., Marsden, B. D. & von Delft, F. PanDDA analysis of BAZ2B screened against Zenobia Fragment Library. (2016) doi:10.5281/ZENODO.48768.

16. Silman, I. & Sussman, J. L. Acetylcholinesterase: 'Classical' and 'non-classical' functions and pharmacology. *Curr. Opin. Pharmacol.* **5**, 293–302 (2005).

17. Sussman, J. L. *et al.* Atomic structure of acetylcholinesterase from Torpedo californica: A prototypic acetylcholine-binding protein. *Science (80-.).* **253**, 872–879 (1991).

18. Alles, G. A. & Hawes, R. C. Cholinesterases in the blood of man.

19. Gilson, M. K. *et al.* Open 'back door' in a molecular dynamics simulation of acetylcholinesterase. *Science (80-.).* **263**, 1276–1278 (1994).

20. Colletier, J. P. *et al.* Structural insights into substrate traffic and inhibition in acetylcholinesterase. *EMBO J.* **25**, 2746–2756 (2006).

21. Bourne, Y. *et al.* Substrate and product trafficking through the active center gorge of acetylcholinesterase analyzed by crystallography and equilibrium binding. *J. Biol. Chem.* **281**, 29256–29267 (2006).

22. Colletier, J. P. *et al.* Shoot-and-trap: Use of specific x-ray damage to study structural protein dynamics by temperature-controlled cryo-crystallography. *Proc. Natl. Acad. Sci. U. S. A.* **105**, 11742–11747 (2008).

23. Owen, R. L., Rudiño-Piñera, E. & Garman, E. F. Experimental determination of the radiation dose limit for cryocooled protein crystals. *Proc. Natl. Acad. Sci. U. S. A.* **103**, 4912–4917 (2006).

24. Ursby, T. & Bourgeois, D. Improved estimation of structure-factor difference amplitudes from poorly accurate data. *Acta Crystallogr. Sect. A* **53**, 564–575 (1997).

25. Dolinsky, T. J., Nielsen, J. E., McCammon, J. A. & Baker, N. A. PDB2PQR: An automated pipeline for the setup of Poisson-Boltzmann electrostatics calculations. *Nucleic Acids Res.* **32**, W665-7 (2004).

26. Nass Kovacs, G. *et al.* Three-dimensional view of ultrafast dynamics in photoexcited bacteriorhodopsin. *Nat Commun* **10**, 3177 (2019).

27. Weik, M *et al.* Specific chemical and structural damage to proteins produced by synchrotron radiation. *Proc. Natl. Acad. Sci. U. S. A.* **97** 623-628 (2000).
